# Cancer associated fibroblast FAK regulates malignant cell metabolism

**DOI:** 10.1038/s41467-020-15104-3

**Published:** 2020-03-10

**Authors:** Fevzi Demircioglu, Jun Wang, Juliana Candido, Ana S. H. Costa, Pedro Casado, Beatriz de Luxan Delgado, Louise E. Reynolds, Jesus Gomez-Escudero, Emma Newport, Vinothini Rajeeve, Ann-Marie Baker, Marina Roy-Luzarraga, Trevor A. Graham, Julie Foster, Yu Wang, James J. Campbell, Rajinder Singh, Penglie Zhang, Thomas J. Schall, Frances R. Balkwill, Jane Sosabowski, Pedro R. Cutillas, Christian Frezza, Patricia Sancho, Kairbaan Hodivala-Dilke

**Affiliations:** 1 Centre for Tumour Biology, Barts Cancer Institute, Queen Mary University of London, John Vane Science Centre, Charterhouse Square, London, EC1M 6BQ UK; 2 Centre for Molecular Oncology, Barts Cancer Institute, Queen Mary University of London, John Vane Science Centre, Charterhouse Square, London, EC1M 6BQ UK; 3 Centre for Cancer and Inflammation, Barts Cancer Institute, Queen Mary University of London, John Vane Science Centre, Charterhouse Square, London, EC1M 6BQ UK; 4 MRC Cancer Unit, University of Cambridge, Hutchison/MRC Research Centre, Cambridge Biomedical Campus, Cambridge, CB2 0XZ UK; 5 Centre for Haemato-Oncology, Barts Cancer Institute, Queen Mary University of London, John Vane Science Centre, Charterhouse Square, London, EC1M 6BQ UK; 6 Centre for Stem Cells in Cancer and Ageing, Barts Cancer Institute, Queen Mary University of London, John Vane Science Centre, Charterhouse Square, London, EC1M 6BQ UK; 7 ChemoCentryx Inc., 850 Maude Ave, Mountain View, CA94043 USA; 8 IIS Aragon, Hospital Universitario Miguel Servet, Zaragoza, 50009 Spain

**Keywords:** Cancer metabolism, Cancer microenvironment, Tumour angiogenesis

## Abstract

Emerging evidence suggests that cancer cell metabolism can be regulated by cancer-associated fibroblasts (CAFs), but the mechanisms are poorly defined. Here we show that CAFs regulate malignant cell metabolism through pathways under the control of FAK. In breast and pancreatic cancer patients we find that low FAK expression, specifically in the stromal compartment, predicts reduced overall survival. In mice, depletion of FAK in a subpopulation of CAFs regulates paracrine signals that increase malignant cell glycolysis and tumour growth. Proteomic and phosphoproteomic analysis in our mouse model identifies metabolic alterations which are reflected at the transcriptomic level in patients with low stromal FAK. Mechanistically we demonstrate that FAK-depletion in CAFs increases chemokine production, which via CCR1/CCR2 on cancer cells, activate protein kinase A, leading to enhanced malignant cell glycolysis. Our data uncover mechanisms whereby stromal fibroblasts regulate cancer cell metabolism independent of genetic mutations in cancer cells.

## Abstract (web summary)

Cancer associated fibroblasts (CAFs) have been suggested to regulate cancer cell metabolism, but the mechanisms are not completely elucidated. Here, the authors show that low FAK expression in stromal cells correlates with poor prognosis in breast and pancreatic cancer patients and that FAK-silencing in CAFs promotes tumourigenesis by the paracrine regulation of cancer cell metabolism.

## Introduction

Understanding the mechanisms that control malignant cell metabolism is a major focus of current cancer research. Previous studies have correlated intrinsic genomic and epigenomic alterations with regulating metabolic features of cancer cells^1,2^. Recent work has demonstrated that the tumour microenvironment, particularly cancer-associated fibroblasts (CAFs), is important in regulating cancer cell metabolism mostly via the production of metabolites that affect cancer cells^3^. However, in vivo evidence dissecting the molecular mechanisms by which CAFs regulate malignant cell metabolism in different tumour models is still required.

Focal adhesion kinase (FAK) is a cytoplasmic non-receptor protein tyrosine kinase and is ubiquitously expressed. Many reports indicate that FAK overexpression in bulk tumour analyses is associated with poor prognosis^4,5^ and these results have spurred the development of FAK inhibitors for cancer treatment. However, other reports suggest either no correlation with prognosis or even that low FAK expression is associated with poor prognosis^6,7^. None of these clinical analyses separate the predictive significance of stromal FAK expression. In mice, genetic dissection of the effect of FAK loss in separate compartments of the tumour stroma have indicated that loss of endothelial cell FAK can affect the initiation of tumour angiogenesis and function depending on the temporal regulation of EC-FAK depletion^8,9^. Additionally, heterozygous depletion of FAK can enhance tumour angiogenesis and tumour growth while loss of haematopoetic FAK can enhance cancer metastasis without an apparent effect on primary tumour growth^10^. The impact of these studies lies in elucidating the multiple functions of FAK in different stromal cell types in the control of cancer growth.

Several reports indicate that fibroblast FAK can induce cell motility, extracellular matrix deposition, survival and proliferation via several signalling pathways in vitro^11,12^ suggesting that FAK expression in CAFs is required for these functions in vivo. In addition to these functions, previously published work has shown that FAK can regulate the production of different chemokines and cytokines depending on the cell type and experimental settings^9,13–15^.

Here, we address the role of FAK in CAFs and show that it regulates tumour cell metabolism by paracrine cytokine signalling. We find that loss of FAK in a subpopulation of CAFs is sufficient to induce increased tumour growth and enhance malignant cell glycolysis. These observations are validated in human breast and pancreatic cancers with low stromal FAK expression. Mechanistically, loss of FAK in a subpopulation of CAFs causes the enrichment of cytokine signalling pathways and the upregulation of Ccl6, Ccl11, Ccl12 and pentraxin-3 resulting in the enhancement of glycolysis in malignant cells. Thus, our study reveals a mechanism of malignant cell metabolism that involves expression of FAK in CAFs.

## Results

### Low FAK expression in the stromal compartment is associated with poor survival in human breast and pancreatic cancers

**Fig. 1:**
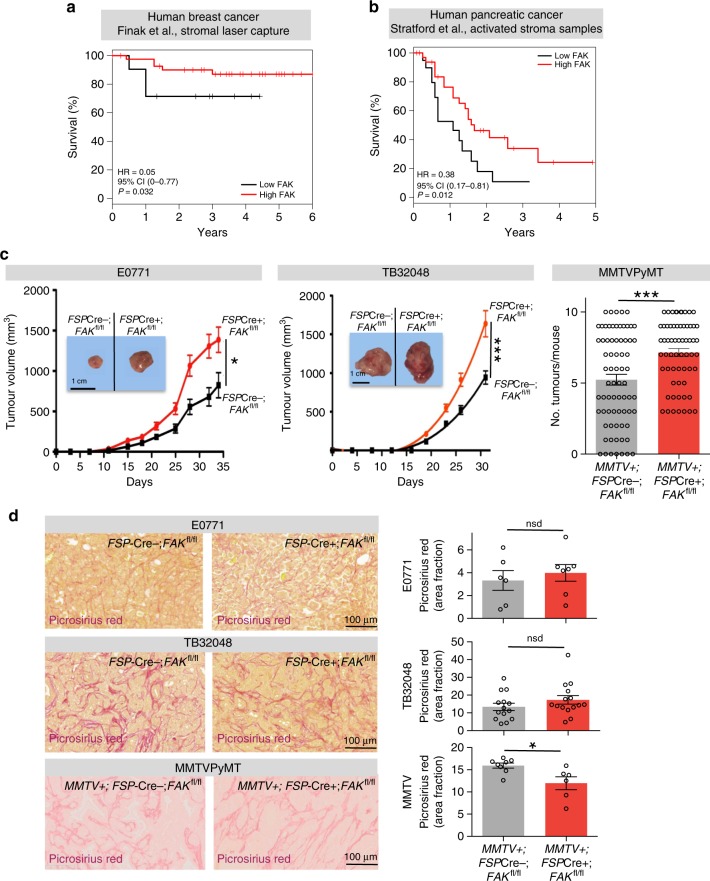
Cancer-associated fibroblast FAK depletion enhances tumour growth. Low stromal FAK expression is significantly associated with reduced overall survival in **a** human breast (microdissected tumour stroma analysis from 53 primary breast tumours, Finak et al. dataset) and **b** human pancreatic cancers (54 human pancreatic cancers with activated stroma, Stratford et al. dataset). The Cox proportional hazards (Coxph) regression analysis was performed, with the rank *P* value shown. See full details in Methods “Gene expression data analysis and clinical inferences” section. **c** Tumour growth is enhanced in *FSP-Cre*+*; FAK*^*fl/fl*^ mice. *FSP-Cre*+*; FAK*^*fl/fl*^ and control *FSP-Cre−;FAK*^*fl/fl*^ mice were injected orthotopically with either syngeneic breast cancer cells (E0771, *n* = 10 *FSP-Cre*+*; FAK*^*fl/fl*^ mice and *n* = 18 *FSP-Cre−;FAK*^*fl/fl*^ mice) or pancreatic ductal adenocarcinoma cells (TB32048, *n* = 10 *FSP-Cre*+*; FAK*^*fl/fl*^ mice and 11 *FSP-Cre−;FAK*^*fl/fl*^ mice). *FSP-Cre*+*; FAK*^*fl/fl*^ and *FSP-Cre−; FAK*^*fl/fl*^ mice were also crossed with MMTV-PyMT mice to generate *MMTV*+*;FSP-Cre*+*;FAK*^*fl/fl*^ and *MMTV*+*;FSP-Cre−;FAK*^*fl/fl*^ mice that developed spontaneous breast tumours. E0771 and TB32048 tumour growth was enhanced in *FSP-Cre*+*;FAK*^*fl/fl*^ mice and the number of tumours per mouse increased significantly in *MMTV*+*;FSP-Cre*+*;FAK*^*fl/fl*^ when compared with control mice. *n* = 11 *MMTV*+*; FSP-Cre*+*;FAK*^*fl/fl*^ and 8 *MMTV*+*;FSP-Cre−;FAK*^*fl/fl*^ mice. Graphs represent mean tumour volume ± s.e.m. Bar chart represents mean no. tumours per mouse ± s.e.m. **d** Picrosirius red staining of late-stage tumour sections from E0711, TB32048 and MMTV-PyMT tumours in *FSP-Cre*+*; FAK*^*fl/fl*^ and *FSP-Cre−; FAK*^*fl/fl*^ mice. Scatter plots represent picrosirius red image analysis (ImageJ) for individual tumours. *n* = 6 *FSP-Cre−; FAK*^*fl/fl*^ and 7 *FSP-Cre*+*; FAK*^*fl/fl*^ E0771 tumours; *n* = 14 *FSP-Cre−; FAK*^*fl/fl*^ and 15 *FSP-Cre*+*; FAK*^*fl/fl*^ TB32048 tumours; *n* = 8 *FSP-Cre−; FAK*^*fl/fl*^ and 6 *FSP-Cre*+*; FAK*^*fl/fl*^ MMTV tumours. Bar chart represents mean ± s.e.m. *P < 0.05, ***P < 0.001. nsd no significant difference. Statistical analysis, two-way ANOVA (**c** for E0771 and TB32048 growth curves); two-sided Student’s *t*-test (**c** for MMTV data, ***P < 0.0002 and **d** *P < 0.05). Scale bars, 1 cm (**c**); 100 μm (**d**).

Increased involvement of stromal CAFs is a pathophysiological feature associated with both breast and pancreatic ductal adenocarcinoma progression but the role of CAF-FAK in progression of these cancers has not been addressed before. Here we performed multivariate analysis of human breast^16^ and pancreatic cancer^17,18^ datasets, and found that low stromal FAK expression in both these cancer types is associated with reduced overall survival (Fig. 1a, b, Supplementary Fig. 1). Given that CAFs represent the major cellular component of the tumour stroma, these data led us to investigate the functional role of low FAK expression in CAFs in cancer growth.

### Development and characterisation of a mouse model of FAK-deletion in CAFs

CAFs are heterogeneous populations of cells and thus no one marker identifies all CAFs^19,20^ To develop a genetic tool to assess if stromal FAK could regulate tumour growth and progression, we used Cre-Lox recombination to delete FAK in the FSP-1-positive subpopulation of CAFs^21^. Please see Methods section for justification for using FSP-Cre+ mice from Gustavo Leone’s laboratory. *FSP-Cre*+*;FAK*^*fl/fl*^ and *FSP-Cre−;FAK*^*flf/fl*^ mice were born at normal Mendelian ratios, and showed no defects in weight, gender distribution and tissue morphology (Supplementary Fig. 2a, b). Primary lung fibroblasts isolated from these mice did not express epithelial and endothelial markers, but did express common markers of fibroblasts, namely, PDGFR-β and FSP-1 (Supplementary Fig. 2c, Supplementary Fig. 7). CAF-specific FAK depletion was confirmed by the following: epithelial cells isolated from breast tumours grown in *MMTV*+*;FSP-Cre*+*;FAK*^*fl/fl*^ or *MMTV*+*;FSP-Cre−;FAK*^*fl/fl*^ mice had no detectable differences in FAK expression levels (Supplementary Fig. 2d, Supplementary Fig 7); using CAG-tdTomato reporter mice, the vast majority (94.8%) of tdTomato-positive cells are CD45 negative (Supplementary Fig. 2e); depletion of FAK was not observed in BMDMs in FSP-Cre+;FAK^fl/fl^ mice (Supplementary Fig. 2f, g, Supplementary Fig 7**)**. Additionally, FSP-1 expression was barely detectable in normal lung fibroblasts from both *FSP-Cre*+*;FAK*^*fl/fl*^ and *FSP-Cre−;FAK*^*fl/fl*^ mice, and its expression was significantly increased after fibroblast activation with a corresponding reduction of FAK only in fibroblasts from *FSP-Cre*+*;FAK*^*fl/fl*^ mice (Supplementary Fig. 2h). Previous reports have indicated that FAK expression can affect the expression of the closely related kinase Pyk2 (refs. ^22–25^) but that compensation is not always evident and depends on the experimental setting^8,24,26^. Here we show that Pyk2 expression was not affected in activated fibroblasts from *FSP-Cre*+*;FAK*^*fl/fl*^ mice (Supplementary Fig. 2h, Supplementary Fig 7). Moreover, depletion of FAK expression was demonstrated in primary CAFs from *MMTV*+*;FSP-Cre*+*;FAK*^*fl/fl*^ mice in vitro and orthotopic pancreatic tumours in vivo (Supplementary Fig. 2i, j, Supplementary Fig 7). Together with published evidence for CAF specificity in *FSP-Cre*+mice^21,27^, our data support depletion of FAK in a subpopulation of CAFs in these *FSP-Cre*+*;FAK*^*fl/fl*^ mice.

### FSP-Cre+;FAK^fl/fl^ mice display increased breast and pancreatic cancer growth

To examine the effects of FAK depletion in FSP-1-positive CAFs on primary tumour growth, syngeneic orthotopic breast and pancreatic cancer growth was assessed using E0771 and TB32048 cells, respectively. Enhanced tumour growth was observed in *FSP-Cre*+*;FAK*^*fl/fl*^ mice for both tumour types. Additionally, these results were supported by an increase in the number of tumours per mouse in *MMTV*+*;FSP-Cre*+*;FAK*^*fl/fl*^ mice compared with controls at week 16 (Fig. 1c, Supplementary Fig. 2k, l). Orthotopic tumour growth was not different in *FSP-Cre*+;non-floxed vs *FSP-Cre*−;non-floxed mice indicating that Cre expression alone had no effect (Supplementary Fig. 2m). Together, these data demonstrate that depletion of FAK in FSP-1-positive CAFs is sufficient to enhance tumour growth and disease progression.

**Fig. 2:**
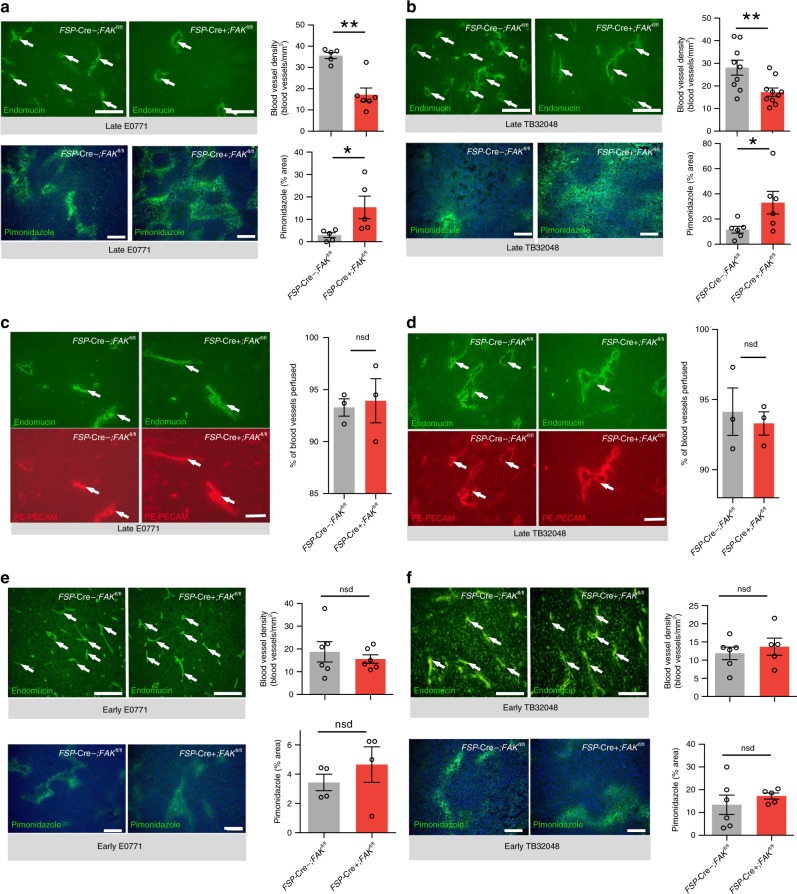
Depletion of CAF-FAK reduces tumour blood vessel density in late stage tumours. Histological analysis of blood vessel density and hypoxia in size-matched, age-matched late stage **a** E0771 (day 34) and **b** TB32048 (Day 33) tumours grown in *FSP-Cre*+*; FAK*^*fl/fl*^ and *FSP-Cre−; FAK*^*fl/fl*^ mice. Tumours were sectioned and immunostained for either endomucin or pimonidazole and the number of blood vessels or relative areas of hypoxia quantified across whole-tumour sections. A significant reduction in blood vessel density and increase in hypoxia were observed in late-stage tumours from *FSP-Cre*+*; FAK*^*fl/fl*^ mice. Bar charts show quantitation of mean blood vessel density per mm^2^ ± s.e.m. and mean pimonidazole positive area fraction ± s.e.m (*n* = 5 (E0771) and 9 (TB32048) *FSP-Cre−; FAK*^*fl/fl*^ and 6 (E0771) and 10 (TB32048) *FSP-Cre*+*; FAK*^*fl/fl*^ tumours, for endomucin; *n* = 5 *FSP-Cre−; FAK*^*fl/fl*^ and *FSP-Cre*+*; FAK*^*fl/fl*^ E0771 tumours and 6 *FSP-Cre−; FAK*^*fl/fl*^ and 5 *FSP-Cre*+*; FAK*^*fl/fl*^ TB32048 tumours, for pimonidazole). **c**, **d** Blood vessel perfusion was measured by calculating the percentage of endomucin-positive blood vessels that were PE-PECAM positive. The functionality of tumour blood vessels was assessed by immunofluorescence staining of endomucin in mice perfused with PE-PECAM antibody on late-stage **c** E0771 breast and **d** TB32048 pancreatic tumour sections. The percentage of perfused blood vessels was not different between the two genotypes. Bar charts show mean percentage of perfused vessels (double positive) over total number of blood vessels (endomucin positive). *n* = 3 tumours from *FSP-Cre−;FAK*^*fl/fl*^ and 3 tumours from *FSP-Cre*+*;FAK*^*fl/fl*^ mice. Arrows, representative perfused vessels. Green: endomucin, red: PE-PECAM. Bar chart in **c** and **d** represents mean % perfused blood vessels ± s.e.m; two-sided Student’s *t*-test. nsd no significant difference. Scale bars, 25 μm. **e**, **f** Before tumour growth diverged significantly between genotypes, early stage, size-matched E0771 breast (day 21) and TB32048 pancreatic (day 21) tumours were also analysed for tumour angiogenesis (E0771: *n* = 6 tumours/genotype; TB32048: *n* = 6 tumours from *FSP-Cre−;FAK*^*fl/fl*^ and 5 tumours from *FSP-Cre*+*;FAK*^*fl/fl*^ mice) and hypoxia (E0771: *n* = 4 tumours/genotype; TB32048: *n* = 6 tumours from *FSP-Cre−;FAK*^*fl/fl*^ and 5 tumours from *FSP-Cre*+*;FAK*^*fl/fl*^ mice) as above. No changes in blood vessel density or hypoxia were detected at this early stage of tumour growth. Bar chart in **e** and **f** shows mean blood vessel density per mm^2^ ± s.e.m. Scale bars in **a**, **b**, **e**, **f** for endomucin, 25 μm; pimonidazole, 200 μm. For **a** and **b**, *P < 0.05, **P < 0.01. nsd, no significant difference. Statistical analysis, two-sided Student’s *t*-test.

We next assessed whether possible changes in components of the tumour microenvironment could inform a cellular basis of the enhanced tumour growth in *FSP-Cre*+*;FAK*^*fl/fl*^ mice. Tumour desmoplasia was assessed by Picrosirius red staining, an indicator of collagen deposition, in late-stage E0771 and TB32048 tumours grown in *FSP-Cre*+*;FAK*^*fl/fl*^, *MMTV*+*;FSP-Cre*+*;FAK*^*fl/fl*^ and control mice. Collagen deposition was unchanged in orthotopic tumours and modestly reduced in breast tumours from *MMTV*+*;FSP-Cre*+*;FAK*^*fl/fl*^ mice (Fig. 1d). These data suggest that FAK expression in FSP-1-positive subpopulation of CAFs has little effect on tumour desmoplasia. This suggests that the increased tumour growth and progression in *FSP-Cre*+*;FAK*^*fl/fl*^ mice does not appear to depend on major changes in desmoplasia. Another component of the tumour stroma is the immune infiltrate and tumour-associated macrophages (TAMs) are known to facilitate tumour growth^28^. Unexpectedly, a significant reduction in TAMs was found in late-stage orthotopic breast and pancreatic tumours grown in *FSP-Cre*+*;FAK*^*fl/fl*^ mice, as well as *MMTV*+*;FSP-Cre*+*;FAK*^*fl/fl*^ mice, compared with control mice whilst no difference was detected in early-stage tumours (Supplementary Fig. 3a–c). No differences were observed in the total numbers and activation of T-lymphocytes, or numbers of B-lymphocytes, dendritic cells and granulocytes (Supplementary Fig. 3d–e). Several studies have indicated that enhanced angiogenesis can induce tumour growth and CAFs are linked to angiogenesis^29^. Thus, we examined blood vessel density and hypoxia in age- and size-matched tumours grown in *FSP-Cre*+*;FAK*^*fl/fl*^ and *FSP-Cre−;FAK*^*fl/fl*^ mice. We showed that blood vessel density was surprisingly decreased with a corresponding increase in tumour hypoxia in both E0771 and TB32048 late-stage tumours (Fig. 2a, b). Given that the number of tumour blood vessels alone is not necessarily indicative of vascular function, we also showed that the perfusion of the tumour blood vessels in *FSP-Cre*+*;FAK*^*fl/fl*^ and *FSP-Cre−;FAK*^*fl/fl*^ mice is similar (Fig. 2c, d). These data indicate that the enhanced tumour growth observed in *FSP-Cre*+*;FAK*^*fl/fl*^ mice was not due to increased tumour angiogenesis or blood vessel perfusion. To address whether this reduction in blood vessel density was a constitutive feature of tumours grown in *FSP-Cre*+*;FAK*^*fl/fl*^ mice, we also examined blood vessel density and hypoxia in early stage, size-matched tumours before the tumour growth diverged significantly between the genotypes. At this stage, both blood vessel density and tumour hypoxia were unchanged in E0771 and TB32048 tumours (Fig. 2e, f). Together, these data suggest that depletion of CAF-FAK enhances tumour growth despite reduced numbers of blood vessels.

### FAK depletion in CAFs enhances malignant cell metabolism

**Fig. 3:**
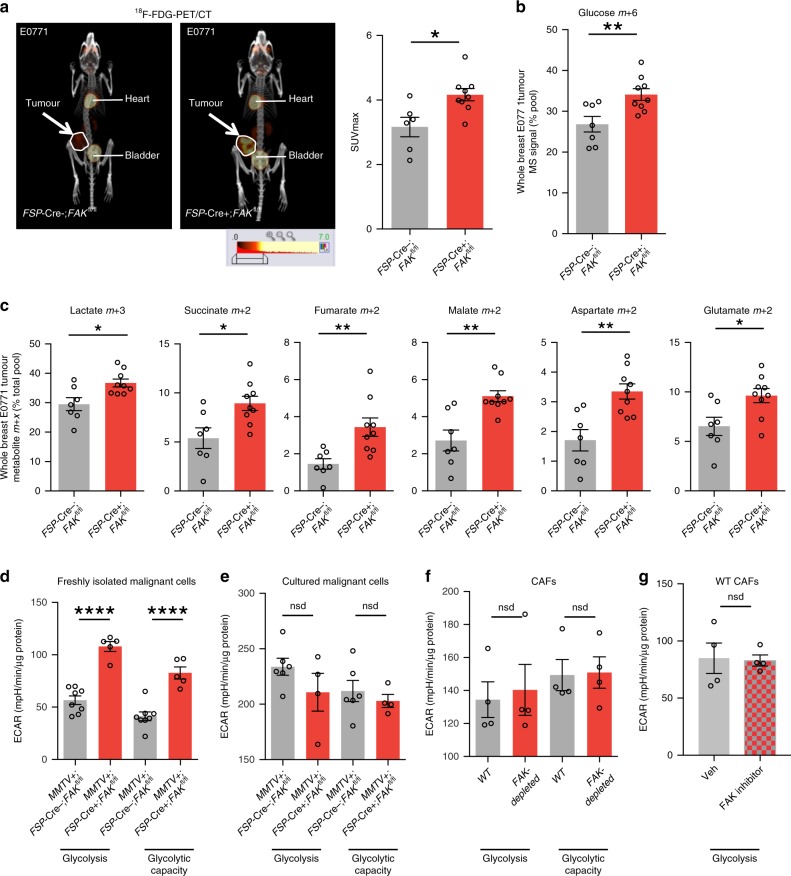
FAK depletion  in FSP-1 positive CAFs alters malignant cell metabolism. **a** ^18^F-FDG PET/CT imaging of early-stage, size-matched E0771 orthotopic breast tumours revealed enhanced glucose uptake in tumours grown in *FSP-Cre*+*;FAK*^*fl/fl*^ mice when compared with controls. PET images expressed on the same scale, range = 0–7 SUV. Bar charts show mean maximum standardised uptake value (SUVmax) ± s.e.m. *n* = 6 *FSP-Cre−;FAK*^*fl/fl*^ and 9 *FSP-Cre*+*; FAK*^*fl/fl*^ mice. **b**, **c** *FSP-Cre*+*; FAK*^*fl/fl*^ and *FSP-Cre−; FAK*^*fl/fl*^ mice bearing early-stage, size-matched E0771 tumours were continuously infused with [U-^13^C6] glucose and LC-MS analysis of metabolites extracted from tumours. Results showed **b** an increase in M+6 isotopologue of glucose, with **c** an enrichment of isotopologues of lactate, succinate, fumarate, malate, aspartate and glutamate from tumours grown in *FSP-Cre*+*;FAK*^*fl/fl*^ mice. Bar charts show mean % of the indicated isotopologue pool over the total metabolite pool ± s.e.m. *n* = 7 *FSP-Cre−;FAK*^*fl/fl*^ and 9 *FSP-Cre*+*; FAK*^*fl/fl*^ tumours. **d** Seahorse extracellular flux analysis of freshly sorted EpCAM+ malignant cells from tumours grown in *MMTV*+*;FSP-Cre*+*;FAK*^*fl/fl*^ mice showed enhanced glycolysis and a significant increase in glycolytic capacity compared with control mice. Bar charts show mean ECAR ± s.e.m. *n* = 3 independent experiments; *n* = 8 *MMTV*+*;FSP-Cre−;FAK*^*fl/fl*^ and 5 *MMTV*+*;FSP-Cre*+*;FAK*^*fl/fl*^ cell technical repeats from a representative run. **e** The changes in glycolysis and glycolytic capacity were lost when malignant cells isolated from *MMTV*+*;FSP-Cre*+*;FAK*^*fl/fl*^ and control mice were isolated and cultured for 3 days; *n* = 3 independent experiments, *n* = 6 *MMTV*+*;FSP-Cre−;FAK*^*fl/fl*^ and 4 *MMTV*+*;FSP-Cre*+*;FAK*^*fl/fl*^ cell technical repeats from the representative run. **f** Primary FAK-depleted and WT-CAFs have similar glycolysis and glycolytic capacity; *n* = 4 independent experiments, 4 technical repeats for each genotype from the representative run. **g** WT-CAFs treated with vehicle alone or FAK-kinase inhibitor (PF PF-573,228) have similar levels of glycolysis. *n* = 4 technical replicates. Bar charts in **e**–**g** represent mean ECAR ± s.e.m. For **a**–**d** *P < 0.05, **P < 0.01, ***P < 0.001, ****P < 0.0001. nsd, no significant difference. Statistical analysis, two-sided Student’s *t*-test.

Cancer cells undergo metabolic changes to support tumour growth and proliferation under harsh environmental conditions^1^. Given the enhanced tumour growth despite reduced blood vessel density, we examined whether the loss of CAF-FAK influenced malignant cell metabolism. ^18^F-FDG-PET/CT imaging of mice with early-stage, size-matched orthotopic breast tumours, which had a similar proliferation index (Supplementary Fig. 4a, b), showed significantly higher SUVmax values indicating a potential increase in glucose uptake in tumours grown in *FSP-Cre*+*;FAK*^*fl/fl*^ mice (Fig. 3a). In line with this result, we detected increased levels of labelled glucose upon infusion of tumour-bearing mice with [U-^13^C6] glucose (Fig. 3b). Further, liquid chromatography-mass spectrometry (LC-MS) analysis demonstrated that in addition to increased lactate levels, glucose significantly increased its contribution to tricarboxyclic acid (TCA) cycle in orthotopic breast tumours. Percentages of labelled isotopologues of TCA cycle intermediates succinate, fumarate and malate as well as aspartate and glutamate were all increased significantly in tumours grown in *FSP-Cre*+*;FAK*^*fl/fl*^ mice (Fig. 3c). Although the increase in lactate levels was not statistically significant, similar findings were detected for TCA cycle intermediates as well as aspartate and glutamate in orthotopic pancreatic tumours grown in *FSP-Cre*+*;FAK*^*fl/fl*^ mice. Furthermore, levels of labelled isotopologues of orotate and uracil were elevated indicating an increase in pyrimidine biosynthesis (Supplementary Fig. 4c). Importantly, these data identified that depletion of FAK in the FSP-1 expressing subpopulation of CAFs alters glucose metabolism in tumours in vivo even before tumour growth rates significantly diverged between *FSP-Cre*+*;FAK*^*fl/fl*^ and *FSP-Cre−;FAK*^*fl/fl*^ mice.

Since LC-MS analysis was performed on whole-tumour extracts, in order to dissect metabolic alterations in different cell types and elucidate the cellular and molecular mechanisms that underlie how reduced expression of CAF-FAK could regulate the metabolism of malignant cells, we compared primary tumour cells from *MMTV*+*;FSP-Cre−;FAK*^*fl/fl*^ and *MMTV*+*;FSP-Cre*+*;FAK*^*fl/fl*^ mammary tumours by Seahorse Extracellular Flux analysis. Freshly isolated malignant cells from *MMTV*+*;FSP-Cre*+*;FAK*^*fl/fl*^ mice had elevated glycolysis and glycolytic capacity when compared with freshly isolated malignant cells from controls (Fig. 3d). Furthermore, these metabolic alterations were lost when primary malignant cells were cultured for 3 days (Fig. 3 e), supporting the idea that the enhanced glycolysis and glycolytic capacity in freshly isolated malignant cells were due to microenvironmental signals. On the other hand, primary CAFs isolated from *MMTV*+*;FSP-Cre*+*;FAK*^*fl/fl*^ and *MMTV*+*;FSP-Cre−;FAK*^*fl/fl*^ mice showed no differences in glycolysis and glycolytic capacity (Fig. 3f). FAK-kinase inhibitor treated WT-CAFs also showed no apparent effect on glycolysis (Fig. 3g). Additionally, FAK-depleted CAFs had increased basal respiration and ATP production when compared with WT-CAFs (Supplementary Fig. 4d), suggesting that they are not primarily supporting tumour cells through lactate to drive increased mitochondrial oxidative phosphorylation^30^. Effects on CAF OCR were also observed when WT-CAFs were treated with the FAK-inhibitor PF-573, 228 (Supplementary Fig. 4e), suggesting that these effects involved the kinase activity of FAK.

**Fig. 4:**
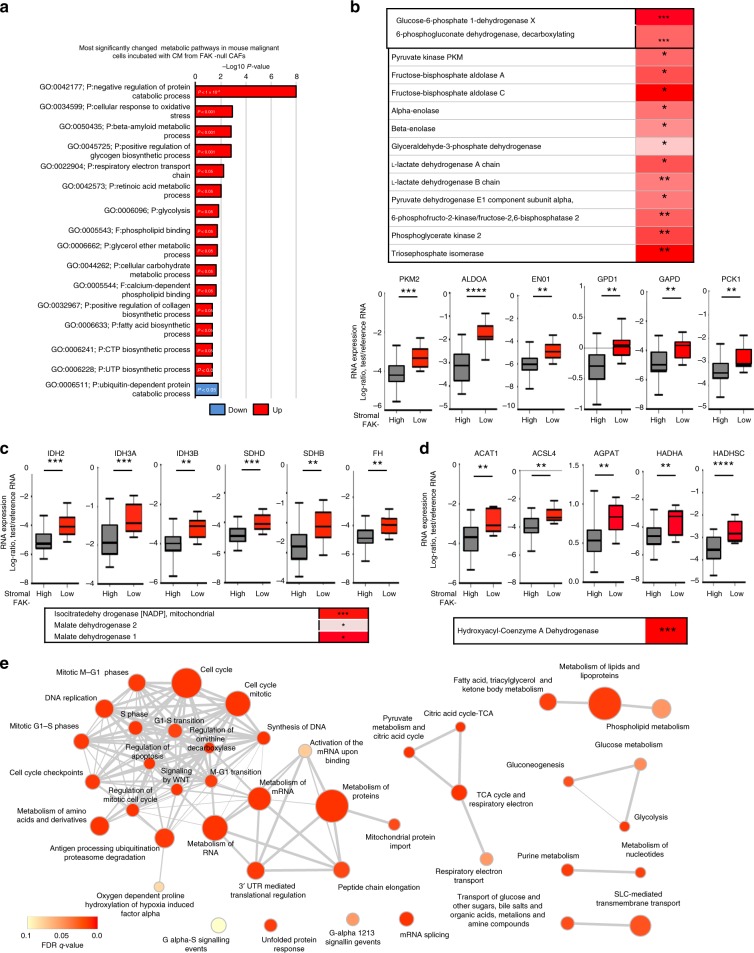
Low stromal FAK expression enhance metabolic pathways in malignant cells. **a** Phosphoproteomics analysis defines a significant enrichment of several metabolic processes in mouse malignant cells exposed to CM from FAK-depleted CAFs compared with malignant cells exposed to WT-CAF CM. **b** Table: the expression levels of several glycolysis enzymes are significantly upregulated (red) in proteomics analysis of mouse malignant cells exposed to FAK-depleted CAF CM when compared with malignant cells exposed to WT-CAF CM. *Box and whisker plots* indicate significant transcriptional upregulation of genes encoding glycolytic enzymes in the cancer cells of patient tumours with low stromal FAK compared with high stromal FAK. **c** *Box and whisker plots*, elevated IDH, SDH and FH gene transcription in the epithelial compartment of patient tumours with low stromal FAK. *Table*: Mouse proteomics data indicate a significant enrichment of IDH peptides in mouse malignant cells exposed to CM from FAK-depleted CAFs compared with malignant cells exposed to WT-CAF CM. **d** *Box and Whisker plots* show elevated transcription of genes encoding enzymes of fatty acid metabolism in the epithelial gene set of human breast cancer with low stromal FAK. *Table*: HADHA upregulation at the protein level in mouse malignant cells exposed to CM from FAK-depleted CAFs. For all human data, *n* = 39 breast cancer patients with high stromal FAK, *n* = 9 breast cancer patients with low stromal FAK (from Finak et al. dataset); for all mouse data *n* = 3 mouse malignant cell preparations exposed to WT-CAF CM and *n* = 3 mouse malignant cell preparations exposed to FAK-depleted CAF CM. Box and whisker plots—box denotes the interquartile range (IQR, Q1 25th percentile—Q3 75th percentile), and whisker denotes the maximum (Q3 + 1.5IQR) and minimum (Q1–1.5IQR). The median value is shown by the horizontal line within the box. *P < 0.05,**P < 0.01,***P < 0.001,****P < 0.0001. nsd, no significant difference. Statistical analysis, two-sided Student’s *t*-test. **e** The transcriptome of cancer cells in breast cancer patients with low vs high stromal FAK were compared using Reactome database. GSEA shows that many pathways regulating cellular metabolism are upregulated in the cancer cells in patient tumours with low stromal FAK expression levels. Disc sizes reflect the number of differentially expressed hits from each pathway. Colour bar indicates FDR *q*-value significance.

Supporting the enhanced malignant cell metabolism described above, proteomics/phosphoproteomics analysis revealed that cellular response to oxidative stress, electron transport chain, glycolysis, fatty acid biosynthesis, CTP and UTP biosynthesis were significantly enriched in primary mouse epithelial cells exposed to FAK-depleted CAF CM (Fig. 4a). Total proteomics analysis revealed a significant increase in the abundance of two key enzymes in the oxidative branch of pentose phosphate pathway (PPP), glucose-6-phosphate dehydrogenase (G6PD) and 6-phosphogluconate dehydrogenase (6PGD) in cancer cells exposed to FAK-depleted CAF CM. PPP is an important source of redox cofactor NADPH and ribonucleotides^31^. Several other glycolytic enzymes, including pyruvate kinase, aldolase, enolase, glycerol 3-phosphate dehydrogenase (GPD), glyceraldehyde 3-phosphate dehydrogenase (GAPD) and phosphoenolpyruvate carboxykinase (PCK), were also elevated (Fig. 4b). Importantly, these changes were validated in the increases of these enzymes at the RNA level in breast cancer patients with low stromal FAK (Fig. 4b, box and whisker graphs). Relevant to the enhanced TCA cycle and electron transport chain, proteomics analysis of mouse epithelial cells exposed to FAK-depleted CAF CM also revealed upregulation of IDH as well as malate dehydrogenase (MDH) 1 and 2 and ATP5B (Fig. 4c, box and whisker graphs). Indeed, isocitrate dehydrogenase (IDH), succinate dehydrogenase (SHD), fumarate hydratase (FH) and ATP5B were all upregulated in human breast cancers with low stromal FAK (Fig. 4c). Similarly, a significant upregulation in the transcription of genes encoding enzymes, which are involved in fatty acid metabolism, were also observed in breast cancer patients and our mouse model (Fig. 4d*)*. Further GSEA of the epithelial compartment using Reactome database identified enrichment in crucial processes for tumour growth and disease progression, such as synthesis of DNA, protein synthesis and cell cycle progression in addition to major metabolic pathways (Fig. 4e). GSEA confirmed significant upregulation of various metabolic pathways, TCA cycle in particular, in the epithelial cell compartment of low-FAK stromal patients using KEGG biological pathways as well (Supplementary Fig. 5a). Together, these data corroborate that low stromal FAK expression regulates malignant cell metabolism in both human and mouse tumours and substantiate the clinical relevance of our findings.

### CAFs control malignant cell metabolism through chemokines Ccl6 and Ccl12

**Fig. 5:**
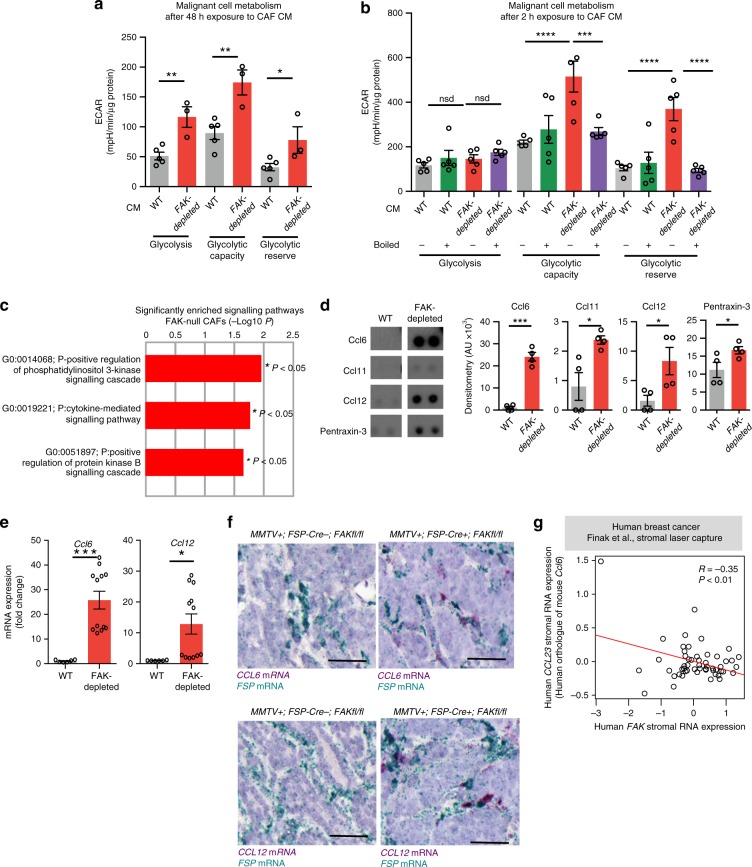
CAF-FAK depletion increases expression Ccl6 and Ccl12. **a** Primary MMTV-derived malignant cells were incubated with the conditioned medium (CM) from primary WT and FAK-depleted CAFs for 48 h. CM from FAK-depleted CAFs enhanced the glycolysis, glycolytic capacity and glycolytic reserve of malignant cells significantly; *n* = 4 independent experiments; *n* = 5 WT CM incubated malignant cells and 3 FAK-depleted CM incubated malignant cells from the representative run. Bar chart shows mean ECAR ± s.e.m. **b** Exposure of malignant cells to CM from FAK-depleted CAFs only for 2 h also increased glycolytic capacity and reserve. Upon boiling the CM for 10 min at 100 °C to inactivate growth factors and cytokines, these metabolic alterations did not persist suggesting a predominantly non-metabolite based communication between CAFs and malignant cells in the regulation of malignant cell metabolism; *n* = 4 independent experiments, 5 technical repeats for each genotype from the representative run. Bar chart shows mean ECAR ± s.e.m. **c** Ontology enrichment analysis of quantitative proteomics data from FAK-depleted CAFs displayed PI3K and cytokine-mediated signalling cascades as the most enriched pathways relative to control WT-CAFs. **d** Proteome profiler arrays identified Ccl6, Ccl11, Ccl12 and Pentraxin-3 as the most upregulated cytokines in primary FAK-depleted CAFs compared with controls; *n* = 4 technical repeats from two independent cell preparations. Bar chart shows mean densitometry (AU) ± s.e.m. **e** qRT-PCR reveals enhanced *Ccl6*, *Ccl12* transcription in FAK-depleted CAF cells; *n* = 2 WT and 4 FAK-depleted cell preps. Bar chart represents mean mRNA expression (fold change) ± s.e.m. **f** RNAscope images of *Fsp-1* and *Ccl6* or *Ccl12* in tumour sections from *MMTV*+*;FSP-Cre−;FAK*^*fl/fl*^ and *MMTV*+*;FSP-Cre*+*;FAK*^*fl/fl*^ mice. Note the increased number of cytokine transcripts (purple) in FSP-1-positive cells (green) in *MMTV*+*;FSP-Cre*+*; FAK*^*fl/fl*^ mice. Scale bars in **f** 50 μm. **g** CCL23, human orthologue of mouse Ccl6, is inversely correlated in human breast cancer patients with stromal FAK expression. *P < 0.05,**P < 0.01,***P < 0.001,****P < 0.0001. nsd, no significant difference. Statistical analysis, **a**–**e** two-sided Student’s test.

We next examined the paracrine mechanism(s) by which CAF-derived signals could affect malignant cell metabolism. Tumour cells, exposed to FAK-depleted CAF CM (48 h), displayed significantly enhanced glycolysis, glycolytic capacity and glycolytic reserve (Fig. 5a) suggesting a paracrine signal from CAFs to malignant cells was sufficient to control malignant cell glycolysis. Reducing the exposure time to 2 h was still sufficient to enhance glycolytic capacity and reserve. However, upon heat inactivation of the conditioned medium, these metabolic alterations were abolished (Fig. 5b), indicating that CAF-FAK regulates the production of proteinaceous factors that can alter cancer cell metabolism. Although these findings would not completely exclude the potential contribution of CAF-derived metabolites, total rescue of metabolic alterations in cancer cells prompted us to focus on the proteome of the CAFs. Proteomics analysis of the primary CAFs revealed a significant enrichment of PI3K and cytokine-mediated signalling pathways in FAK-depleted CAFs (Fig. 5c). Comparison of the secretomes using cytokine arrays (mouse proteome profiler dot blot arrays) showed that Ccl6, Ccl11, Ccl12 and Pentraxin-3 were significantly upregulated in FAK-depleted CAFs (Fig. 5d, Supplementary Fig. 5b, c*)*. Additionally, an increase in Ccl6 and Ccl12 mRNA was observed in FAK-depleted CAFs (Fig. 5e), and further analysis for chemokines, which were not present in the array, revealed that two other CC chemokines, Ccl7 and Ccl8, were also transcriptionally upregulated in FAK-depleted CAFs (Supplementary Fig. 5d). In vivo evidence of this upregulation of chemokines was also found since *Ccl6* and *Ccl12* transcript levels were upregulated in FSP-1-positive CAFs in tumour sections from *MMTV*+*;FSP-Cre*+*;FAK*^*fl/fl*^ compared with control mice, demonstrating their regulation by CAF-FAK in vivo **(**Fig. 5f). Human orthologues of mouse *Ccl6* and *Ccl7*, namely *CCL23* (ref. ^32^) and *CCL7*, respectively, were shown to be inversely correlated with stromal FAK expression in breast cancer patients providing human relevance of our findings (Fig. 5g, Supplementary Fig. 5e).

FAK has both kinase and scaffolding functions and FAK-kinase inhibitors are currently being tested in clinical trials for cancer treatment raising the question as to whether such inhibitors might affect chemokine production in CAFs. Treatment of WT-CAFs with the FAK-kinase inhibitor, PF-573,228, was sufficient to enhance Ccl6, Ccl11 and pentraxin protein expression levels but to a lesser extent than found in FAK-depleted CAFs. However, Ccl12 protein expression levels were not increased by inhibition of FAK kinase activity suggesting that other non-kinase FAK-mediated mechanisms control this chemokine (Supplementary Fig. 5f). Indeed, FAK-kinase inhibition was sufficient to enhance *Ccl6*, but not *Ccl12*, transcription in WT-CAFs. Given that the PI3K signalling pathway is significantly upregulated in FAK-depleted CAFs and this pathway has been previously shown to influence the production of inflammatory cytokines^33^, we assessed *Ccl12* mRNA levels in FAK-depleted CAFs after treatment with the pan-class I PI3K inhibitor GDC-0941. PI3K inhibition significantly downregulated *Ccl12* transcription compared with untreated FAK-depleted CAFs suggesting that *Ccl12* and *Ccl6* expression are likely regulated by different mechanisms (Supplementary Fig. 5g). Together, these data indicate that FAK depletion in CAFs can increase the expression of several chemokines and suggest that chemokine regulation involves both FAK-kinase and non-kinase mediated mechanisms.

### FAK depletion in CAFs increases chemokine production, which via CCR1/CCR2 on cancer cells, activate protein kinase A, leading to enhanced malignant cell glycolysis

**Fig. 6:**
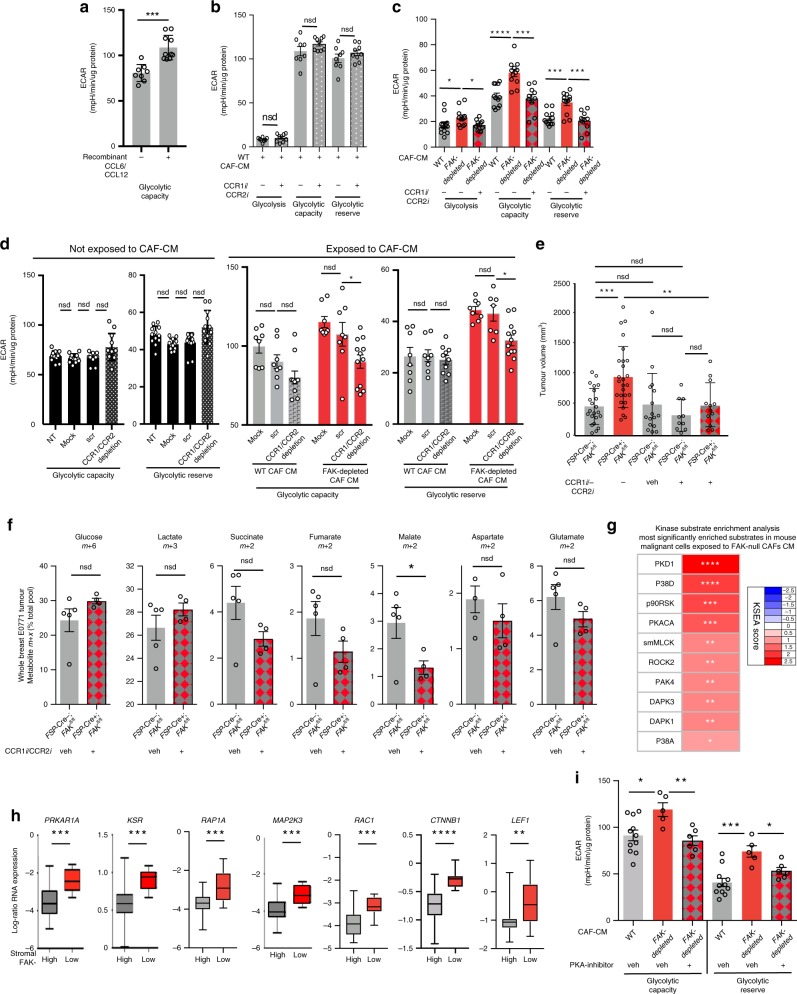
CCR1/2 or PKA inhibition rescues the enhanced metabolism in malignant cells. **a** Treatment of epithelial cells isolated from *MMTV*+*;FSP-Cre−;FAK*^*fl/fl*^ mice with recombinant Ccl6 and Ccl12 for 5 h increases glycolytic capacity in extracellular flux analysis; *n* = 8 non-treated and 10 recombinant Ccl6 and Ccl12 epithelial cell treated technical replicates. **b** Extracellular flux analysis of malignant cells exposed to WT-CAF CM with or without CCR1*i*/CCR2*i* for 48 h shows that these inhibitors have no apparent effect on glycolysis, glycolytic capacity or glycolytic reserve; *n* = 8 biological repeats-CCR1*i/*CCR2*i*, *n* = 9 biological repeats + CCR1*i/*CCR2*i*. **c** Extracellular flux analysis of epithelial cells isolated from *MMTV*+*;FSP-Cre;FAK*^*fl/fl*^ mice treated with CM from either WT or FAK-depleted CAFs in the absence or presence of the dual CCR1*i*/CCR2*i* shows that dual inhibition of CCR1 and CCR2 is sufficient to rescue the metabolic alterations in cancer cells treated with FAK-depleted CAF CM; *n* = 3 independent experiments; *n* = 12 WT-CAF-CM—CCR1*i*/CCR2*i*, *n* = 12 FAK-depleted CAF-CM—CCR1*i*/CCR2*i*, *n* = 10 FAK-depleted CAF-CM+CCR1*i*/CCR2*i*. **d** Effect of CCR1/CCR2- siRNA depletion on breast cancer cell E0771 metabolism. Without exposure to CAF-CM, under normal culture conditions, CCR1/CCR2 depletion in E0771 cells (*n* = 10 glycolytic capacity; *n* = 12 glycolytic reserve) does not affect glycolytic capacity or glycolytic reserve compared with no-treatment (NT) (*n* = 12), mock (*n* = 12) or scrambled (scr) (*n* = 12) negative controls. CCR1/CCR2 depletion in E0771 cells had no significant effect on glycolytic capacity (*n* = 10) or glycolytic reserve (*n* = 10) after exposure to WT-CAF CM, but significantly reduced the enhanced glycolytic capacity (*n* = 12) and glycolytic reserve (*n* = 12) after exposure to FAK-depleted CAF CM. Statistics, two-way ANOVA. Bar charts in **a**–**d** show mean ECAR ± s.e.m. **e** E0771 tumour volumes, at day 28 post tumour cell inoculation in *FSP-Cre−;FAK*^*fl/fl*^ and *FSP-Cre*+*;FAK*^*fl/fl*^ mice. Cohorts: Untreated *FSP-Cre−;FAK*^*fl/fl*^ mice (*n* = 22 mice); untreated *FSP-Cre*+*;FAK*^*fl/fl*^ mice (*n* = 26 mice); vehicle-treated *FSP-Cre−;FAK*^*fl/fl*^ mice (*n* = 15 mice); CCR1*i*/CCR2*i*-treated *FSP-Cre−;FAK*^*fl/fl*^ mice (*n* = 10 mice) and CCR1*i*/CCR2*i*-treated *FSP-Cre*+*;FAK*^*fl/fl*^ mice (*n* = 12 mice). Bar chart shows mean tumour volume (mm^3^) ± s.e.m. **f** *FSP-Cre*+*; FAK*^*fl/fl*^ mice treated with CCR1*i*/CCR2*i* and vehicle-treated *FSP-Cre−; FAK*^*fl/fl*^ mice, both bearing early-stage, size-matched E0771 tumours, were continuously infused with [U-^13^C6] glucose and LC-MS analysis of whole tumours was performed. Inhibition of CCR1/CCR2 reduced the levels of isotopologues of glucose, lactate, succinate, fumarate, malate, aspartate and glutamate from tumours grown in *FSP-Cre*+*;FAK*^*fl/fl*^ mice. Bar charts show mean % of the indicated isotopologue pool over the total metabolite pool ± s.e.m. *n* = 4 *FSP-Cre−; FAK*^*fl/fl*^ and 5 *FSP-Cre*+*; FAK*^*fl/fl*^ tumours. **g** Kinase substrate enrichment analysis (KSEA) of phosphoproteomics data showed a significant enrichment of PKD1, p38δ, p90RSK, PKACA, ROCK2, PAK4, DAPK3, DAPK1, p38α and MLCK substrates in cancer cells exposed to CM from FAK-depleted CAFs; *n* = 3 independent cell lysates for each genotype, two-sided Student’s *t*-test. **h** Epithelial transcript levels of the genes encoding *PRKAR1A*, *KSR*, *RAP1A*, *MAP2K3*, *RAC1*, *CTNNB1* and *LEF1* are significantly enriched in human breast cancer gene set of cancers with low stromal FAK levels; Box and whisker plots show log-ration mRNA expression and include maximum and minimun data points, median value and 75th and 25th quartile. *n* = 39 patients with high stromal FAK and 9 patients with low stromal FAK, Student’s *t*-test. **i** Seahorse analysis of epithelial cells exposed to CM from WT-CAFs and FAK-depleted CAFs plus the PKA inhibitor, KT 5720 (+) or vehicle alone (veh) for 3 h show that treatment with PKA inhibitor reduces the enhanced glycolytic capacity and glycolytic reserve in primary tumour cells exposed to CM from FAK-depleted CAFs that of tumour cells exposed to WT-CAF CM. Bar chart shows mean ECAR ± s.e.m. *n* = 2 cell preparations, scatter dots represent *n* = 11 WT-CAF CM- veh, *n* = 5 FAK-depleted CAF-CM- veh, *n* = 6 FAK-depleted CAF-CM+ inhibitor technical repeats from one representative run. *P < 0.05, **P < 0.01, ***P < 0.001, ****P < 0.0001. nsd, no significant difference. Statistical analysis, two-sided Student’s *t*-test unless otherwise stated.

To examine the molecular mechanisms that control elevated metabolism in malignant cells exposed to a FAK-depleted CAF environment, we turned our attentions to signalling downstream of chemokine stimulation and especially Ccl6 and Ccl12 since these were the most highly upregulated. The functional roles of Ccl6 and Ccl12 in the regulation of malignant cell metabolism were supported by several lines of evidence. Firstly, the addition of recombinant Ccl6 and Ccl12 to the culture medium of breast tumour cells was sufficient to enhance their glycolytic capacity (Fig. 6a) supporting the notion that these chemokines are capable of altering malignant cell metabolism. Secondly, in order to test whether the receptors for these chemokines, namely CCR1 and CCR2 (ref. ^34^), are involved in regulating cancer cell metabolism, malignant cells from *MMTV*+*;FSP-Cre−;FAK*^*fl/fl*^ mice were exposed to WT-CAF CM, FAK-depleted CAF CM or FAK-depleted CAF CM with inhibitors to these receptors, CCR1*i*/CCR2*i*, or vehicle alone. Results revealed that the addition of CCR1*i*/CCR2*i* into WT-CAF CM had no effect on cancer cell metabolism (Fig. 6b), likely because WT-CAFs did not have high levels of expression of Ccl6 or Ccl12. In contrast, the enhanced glycolytic capacity and glycolytic reserve in cancer cells exposed to FAK-depleted CAF CM were significantly reduced by treatment with CCR1*i*/CCR2*i* (Fig. 6c). Thirdly, siRNA knockdown of CCR1 and CCR2 in E0771 breast cancer cells had no significant effect on metabolism under normal culture conditions, but significantly inhibited glycolytic capacity and glycolytic reserve when malignant cells were exposed to FAK-depleted-CAF CM, but not WT-CAF CM (Supplementary Fig. 5h, Fig. 6d). These data suggested that pharmacological or genetic depletion of CCR1 and CCR2 do not affect breast cancer cell metabolism under normal culture conditions but only after exposure to FAK-depleted CAF CM. Indeed, CCR1*i*/CCR2*i* treatment had no effect on tumour burden in *FSP-Cre−;FAK*^*fl/fl*^ mice compared with untreated or vehicle alone treated *FSP-Cre−;FAK*^*fl/fl*^ mice. In contrast, CCR1*i*/CCR2*i* treatment significantly reduced the elevated tumour burden in *FSP-Cre*+*;FAK*^*fl/fl*^ mice down to those in *FSP-Cre−;FAK*^*fl/fl*^ control mice (Fig. 6e). These results correlated with a rescue in the metabolic phenotype in these tumours, where LC-MS analysis demonstrated that treatment of *FSP-Cre*+*;FAK*^*fl/fl*^ mice with CCR1*i*/CCR2*i* reduced bulk tumour metabolite levels down to those found in vehicle-treated *FSP-Cre−;FAK*^*fl/fl*^ control mice (Fig. 6f). Of note, CCR1 and CCR2 are members of the family of G-protein coupled receptors (GPCR)^35^ and enhanced GPCR signalling through Gα_s_ and Gα_12/13_ was also observed in the epithelial compartment of patient tumours with low stromal FAK correlating low stromal FAK expression, cancer progression, enhanced metabolism with elevated signalling via GPCRs in human cancer (see Fig. 4e*)*. Together, these cross-supporting data provide evidence that CCR1 and CCR2 are functionally involved in the enhanced glycolysis of cancer cells after exposure to a FAK-depleted CAF environment.

It has been reported that CCR1 and CCR2 can influence the myeloid infiltrate^36,37^. The reduction in TAMs in late-stage tumours in *FSP-Cre*+*;FAK*^*fl/fl*^ mice prompted us to investigate the metabolic phenotype of these cells. Although BMDMs from non-tumour-bearing *FSP-Cre*+*;FAK*^*fl/fl*^ and control mice show no differences in metabolism, BMDMs from tumour-bearing *MMTV*+*;FSP-Cre*+*;FAK*^*fl/fl*^ mice display significantly elevated glycolysis and glycolytic capacity compared with those from *MMTV*+*;FSP-Cre−;FAK*^*fl/fl*^ mice (Supplementary Fig. 6a, b). Furthermore, exposure of BMDMs to FAK-depleted CAF CM for 3 h increased glycolytic capacity and glycolytic reserve significantly **(**Supplementary Fig. 6c) suggesting that although FAK-depleted CAF CM is sufficient to induce modest metabolic alterations in macrophages, tumour cell-derived factors are very likely involved in enhanced glycolysis in BMDMs upon depletion of FAK in CAFs.

Further investigation of the molecular control of malignant cell metabolism regulation, using Kinase Substrate Enrichment Analysis (KSEA)^38^ in cancer cells exposed to FAK-depleted CAF-CM, identified substrate enrichment in multiple signalling pathways that included protein kinase A catalytic subunit α (PKACA), PKD1, p38δ, p90RSK, smMLCK, ROCK2, PAK4, DAPK3, DAPK1 and p38α, all known to be involved, directly or indirectly, in enhanced cell metabolism (Fig. 6g). Although many chemokine receptors are coupled to G_αi_ subunit, which inhibits adenylate cyclase activity and cAMP production^35^, we detected enrichment of PKA substrates. For example, PKD1, a substrate of PKA^39^, stimulates p42/44 MAPK (Ras–Raf–MEK–Erk) signalling pathway^40^ subsequently enhancing p90RSK, an important regulator of protein synthesis^41^ and glycolysis^42^. Stress activated p38MAPK signalling pathway was also upregulated in cancer cells through p38α and p38δ, which is known to be involved in glucose metabolism through PKD1^43^. Additionally, substrates for PAK4 (p21(RAC1) activated kinase 4), a regulator of glucose intake, NADPH production and lipid synthesis through its interaction with glucose-6-phosphate dehydrogenase (G6PD)^44^ and ROCK2 were also enhanced. ROCK2 is downstream of RhoA from Rho family of GTPases and is activated through coupling of G proteins to Rho guanine nucleotide exchange factors^45^. KSEA analysis identified similar substrate enrichment for Ca^2+^/calmodulin-dependent serine threonine kinases MLCK and DAPK1 (Fig. 6g) supporting the activation of GPCR signalling. A direct link between these kinases, which are mainly involved in cystoskeletal remodelling, and cellular metabolism has not been identified to date. Additionally, stimulation of GCPRs can result in the release of Ca^2+^ from intracellular stores through the generation of diacylglycerol and inositol 1,4,5-triphosphate (IP_3_)^46^. IP_3_ receptor ITPR3 phosphorylation at serine 1832 residue, which is a PKA phosphosite^47^, was enhanced in cancer cells treated with CAF-depleted CM in addition to calmodulin and phosphorylated calcium/calmodulin-dependent protein kinase kinase 2 (CaMKK2). Ca^2+^/calmodulin/CaMKK2 axis also influences glucose and lipid metabolism^48^ (Supplementary Fig. 5i). Wnt/beta-catenin signalling regulates metabolic reprogramming in the cancer cells as it upregulates cMyc expression in a TCF-dependent manner^49^. We observed enhanced phosphorylation of beta-catenin at PKA site serine 552 that promotes TCF/LEF transactivation^50^ (Supplementary Fig. 5i). Additionally, phosphoCTNNA1(alpha-catenin)(serine 641) was additionally upregulated supporting beta-catenin transactivation^51^. In line with the integration of JAK/STAT and G protein pathways upon chemokine exposure, total STAT3 and phosphoSTAT3 (Y705) levels were enhanced in cancer cells exposed to FAK-depleted CAF CM^52^ (Supplementary Fig. 5i). The JAK/STAT3 pathway is also a known mediator of glucose^53^ and fatty acid metabolism^54^. The clinical relevance of these signalling changes was demonstrated by the elevated transcription of genes encoding the regulatory subunit of PKA (PRKAR1A), Rap1A, Kinase suppressor of Ras (KSR), MAP2K3 (MKK3), small GTPase Rac1, CTNBB1 (beta-catenin) and LEF1 in the epithelial compartment of human breast cancers with low levels of stromal FAK (Fig. 6 h). While Rap1-KSR interaction downstream of PKA sustains Erk activation^55^, MAP2K3 (ref. ^56^) is important for the activation of p38 MAPKs. Together, these data identify several putative metabolism regulatory pathways that are enhanced in cancer cells exposed to a FAK-depleted CAF environment.

Since PKA can interact with many signalling alterations directly or indirectly observed in phosphoproteomics analysis, we treated malignant cells isolated from *MMTV*+*;FSP-Cre−;FAK*^*fl/fl*^ mice with conditioned medium from FAK-depleted CAFs plus or minus a pharmacological inhibitor of PKA (KT 5720) for the functional validation of these signalling changes. Seahorse analysis demonstrated that PKA inhibition was sufficient to reduce the enhanced glycolytic capacity and glycolytic reserve of malignant cells exposed to FAK-depleted CAF CM back to that of vehicle-treated cancer cells after exposure to conditioned medium from WT-CAFs (Fig. 6i). DAG and intracellular calcium release enhances protein kinase C activity upon activation of GPCRs. Since PKA and PKC signalling may converge through common downstream effectors, we also tested the effect of PKC inhibition on the same metabolic parameters. Addition of PKC inhibitor (Go6983) to the CM, at a concentration of 10 μM, which inhibits PKCα, β,γ, δ isoforms, did not rescue the enhanced glycolytic capacity and reserve (Supplementary Fig. 6d). Although pharmacological inhibitors can have off-target effects, the validation of the specificity and the efficacy of the pharmacological inhibitors used in this study are provided (Supplementary Fig. 6e–h, Supplementary Fig 7).

Together, our data indicate that the elevated production of Ccl6 and Ccl12 from FAK-depleted CAFs activates chemokine receptors CCR1 and CCR2 on malignant cells altering the regulation of downstream signals that control malignant cell metabolism. Indeed, the enhanced activation of malignant cell PKA after exposure to FAK-depleted CAF CM is required for the upregulation of malignant cell metabolism.

## Discussion

Overall, our data identify in vivo and in vitro evidence by which CAFs regulate malignant cell metabolism. We show that reduced FAK expression levels in FSP-1-positive subpopulation of CAFs are sufficient to induce alterations in signalling and increase tumour growth by enhancing discrete metabolic pathways in cancer cells of the same oncogenic profile.

Our findings indicate that CAF-FAK expression regulates the expression of chemokines Ccl6 and Ccl12, which through malignant cell Ccr1/Ccr2 activity and PKA activation can control cancer cell metabolism. The data establish a concept in CAF-FAK-regulated and chemokine-mediated control of cancer metabolism with relevance to human breast cancers with low stromal FAK expression, and identify potential novel actionable targets for anticancer therapy. Although growth factors have been the focus of extracellular stimuli that initiate signal transduction, induce entry into cell cycle and reprogramme the metabolism to fulfil the biosynthetic needs of cell growth and division^3^, our study demonstrate that chemokines can also play similar roles to support cancer cells and notably enhance malignant cell metabolism.

Our major focus has been on the control of cancer cell Ccr1 and Ccr2 in CAF-FAK regulation of malignant cell metabolism as we detected the most significant increases in their ligands, Ccl6 and Ccl12, respectively. However, additional CC chemokines including Ccl7, Ccl8 and Ccl11, which can bind to other chemokine receptors, are also upregulated in FAK-depleted CAFs. Considering that their associated receptors all belong to the same family of GPCRs, it is plausible that additional chemokine–receptor interactions contribute to the changes in the signalling and metabolic networks that we have observed. Moreover, GPCR coupling diversity can lead to distinct functional outcomes as different receptors can selectively couple to multiple subtypes of G proteins, exhibit differential engagement of various kinases and recruit diverse conformational ensembles of beta-arrestins^57^. Receptor heterodimerisation and the relative abundance of different chemokines would also very likely contribute to diversity in biased signalling in this context. It is also plausible that activation of these major signalling pathways alter the secretome of cancer cells in FAK-depleted environment leading to additional changes in autocrine signalling. Our data point towards activation of putative signalling pathways downstream of chemokine receptors. Most of these pathways can directly be regulated by PKA in addition to PKA- and G protein-independent mechanisms and contribute to distinct metabolic alterations in cancer cells within a FAK-depleted stromal microenvironment.

Previous reports show that FAK regulation of cytokine production is cell type-dependent and also influenced by the experimental conditions^9,13–15^. Reconciling the different effects of FAK loss in different cell types in a whole organism likely reflects a combination of differences in cell functions, intrinsic molecular profiles and local environmental stimuli in combination with different effects on cytokine production. Our data indicate that loss of FAK in CAFs from *FSPCre−;FAK*^*fl/fl*^ mice increases Ccl6, Ccl11, Ccl12 and pentraxin-3 expression with an enhancement in malignant cell glycolysis and tumour growth. This control of chemokine production aligns with work where Col1a-Cre driven loss of FAK expression in activated fibroblasts from a hypertrophic scar formation model increases Ccl2 expression^13^. Conversely, although loss of FAK in endothelial cells of established tumours had no effect on tumour blood vessel density, DNA-damaging agent treatment of FAK-null endothelial cells induced lower amounts of multiple interleukins, thus enhancing malignant cell chemosensitivity and reducing tumour growth^9^. Future investigations will determine the common and distinct molecular mechanisms by which FAK controls cytokine production in different stromal cell types.

FAK has both kinase and scaffold functions and although FAK-kinase inhibitors have shown promise in preclinical studies^14,15^ the outcome of FAK inhibition in initial phase II COMMAND-A trial clinical trial or phase II trial using the combination of FAK inhibitor (GSK2256098) with Trametinib have not been as encouraging as hoped for^58,59^. Further clinical trials using FAK-inhibitors and combination with other therapies, including immunotherapies, are predicted to be more promising and will determine the full utility of FAK inhibitors across multiple cancer types^15^. Since FAK has both kinase and non-kinase functions, it is fair to say that the effect of reduced FAK expression levels and FAK kinase inhibition may not be identical. The balance between signalling alterations due to direct effects of FAK inhibition in cancer cells and stromal fibroblasts could potentially be important to determine the overall treatment outcome.

Our study points towards CAF-mediated mechanisms leading to reduced overall survival in breast and pancreatic cancer patients with low stromal FAK expression and highlight possible metabolic/signalling pathways that could be targeted in these patients.

## Methods

### Generation of mice

Female FAK floxed mice (C57/BL6) were bred with FSP-1 Cre male mice (C57/BL6, originally developed by Prof Gustavo Leone, Cleveland Ohio, USA)^21,60,61^ to generate *FSP-Cre−;FAK*^*fl/fl*^ and *FSP-Cre*+*;FAK*^*fl/fl*^ mice. *MMTV-PyMT*+*;FSP-Cre*+*;FAK*^*fl/fl*^ were also generated by crossing *MMTV-PyMT*+ mice with *FSP-Cre*+*;FAK*^*fl/fl*^ mice.

In our study, we used the FSP-1-Cre mice developed by Gustavo Leone, Cleveland Ohio, USA. These FSP-Cre+ mice display Cre activity specific in a subpopulation of activated fibroblasts and CAFs. FAK expression levels were not affected in epithelial cells and macrophages isolated from *MMTV-PyMT*+*;FSP-Cre*+*;FAK*^*fl/fl*^ derived tumours indicating no effect in these cell types (Please see Extended Fig. 2d, f, g).

To clarify, we have not used FSP-Cre mice from JaxLabs in this study. Although, JaxLabs also sell an FSP-Cre mouse line, these mice show poor specificity to fibroblasts or CAFs, and have also been shown to induce deletion of target genes in other cell types including epithelial cells and macrophages. The reason for the differences in the FSP-Cre+ transgenic from JaxLab and Leone’s laboratory are likely to be related to the fact that they were generated independently by transgene insertion of different sequences of the FSP promoter regions.

For animals bred in-house, health screens (quarterly) were conducted in accordance with FELASA guidelines for health monitoring of rodent colonies, to confirm their free status of known pathogens in accordance with FELASA screens. No clinical signs were detected. Animals were housed in groups of 4–6 mice per individually ventilated cage in a 12 h light dark cycle (06:30–18:30 light; 18:30–06:30 dark), with controlled room temperature (21 ± 1 °C) and relative humidity (40–60%). The cages contained 1–1.5 cm layer of animal bedding, and with environmental enrichment including cardboard Box-tunnel and crinkled paper nesting material. Animals had access to food and water ad libitum.

### Cell culture

E0771 murine breast cancer cells (derived from C57/BL6) were obtained from Prof Anne Ridley (Bristol University, UK) and grown in RPMI medium supplemented with 10% FBS and 1% penicillin streptomycin. *Kras*^*LSL.G12D/+;*^ *p53*^*R172H/+*^*;PdxCre*+ (KPC)-derived TB32048 murine pancreatic cancer cell line (derived from C57/BL6) was obtained from Prof David Tuveson (Cold Spring Harbour, USA) and cultured in DMEM supplemented with 10% FBS and 1% penicillin streptomycin. Primary mouse lung fibroblasts and MMTV-derived CAFs were cultured in DMEM supplemented with 10% FBS, 1% Insulin-Transferrin-Selenium (ITS) supplement and 1% penicillin streptomycin. MMTV-derived primary tumour cells were cultured in Advanced DMEM/F12 supplemented with 2% FBS, 1% penicillin streptomycin, GlutaMAX, Insulin and EGF (20 µg/µL) and on collagen-coated dishes.

### Isolation of CAFs and cancer cells from MMTV-PyMT tumours

CAFs were isolated from *MMTV*+*;FSP-Cre−;FAK*^*fl/fl*^ and *MMTV*+*;FSP-Cre*+*;FAK*^*fl/fl*^ mice as described previously^62^. Tumours were cut into small pieces and digested for 1 h at 37 °C in 5 mL collagenase/dispase (1 mg/mL; Sigma). After filtering the undigested tissue, the solution was centrifuged (3000 *g*, 5 min) and the final pellet was resuspended in DMEM high glucose, 10% FBS, 1% ITS and seeded on a 6 cm dish. After 30 min, fibroblasts are adhered and non-adherent tumour cells were replated and cultured as described above. Adherent fibroblasts were used after 1–3 passages.

### Intrapancreatic injection

Eight- to 10-week-old male mice were anaesthetised with isoflurane. The abdominal skin and muscle were incised just off the midline and directly above the pancreas to allow visualisation of the pancreatic lobes; the pancreas was gently retracted and positioned to allow for a direct injection of 10 µL of 1 × 10^3^ TB32048 cells in PBS-Matrigel (1:1) using a Hamilton syringe. The pancreas was placed back within the abdominal cavity. Both the muscle and skin layers were closed with 6-0 silk sutures (Ethicon). Mice were kept on a heat box during the entire procedure.

### Mammary fat pad injection

Eight- to 10-week old female mice were anaesthetised with isoflurane. A vertical incision was made through the abdominal skin, but not the peritoneum. The skin was pulled away from the peritoneum to expose the fourth inguinal mammary gland and 4 × 10^5^ E0771 tumour cells were injected unilaterally in 20 µL of PBS-matrigel using a 28-gauge needle.

### Immunohistochemistry and immunofluorescence for endomucin and quantification of blood vessel density

Tissues were fixed in formalin for 24 h and transferred to 70% ethanol. Tissues were paraffin embedded, sectioned, dewaxed, and antigen retrieval performed by boiling in 10 mM citrate buffer pH 6.0. Sections were washed three times in PBS, blocked in 5% normal goat serum for 1 h, and incubated with primary antibody rat monoclonal endomucin (1:100 dilution; Santa Cruz, #sc-65495) overnight at 4 °C. Sections were then washed and incubated for 45 min with secondary fluorophore (1:100; AlexaFluor 488 goat anti-rabbit IgG; LifeTechnologies, #A11008) for endomucin staining and finally mounted with Prolong antifade with 4′,6-diamidino-2-phenylindole (DAPI; Molecular Probes). Images were taken with an Axioplan Zeiss microscope. To quantify the tumour blood vessel density, the number of endomucin-positive blood vessels present across the entire area of each midline tumour section from size and age-matched tumours was counted and divided by the area of the section.

### Picrosirius Red staining

For collagen staining, sections were dewaxed and hydrated before staining in Picrosirius Red solution (DirectRed 80; Sigma-Aldrich, #365548) for 1 h. The sections were washed twice in acidified water and then mounted for microscopy. Positive fraction of area (%) of red staining of the entire section was quantified after setting up a threshold using ImageJ.

### Immunohistochemistry using the Ventana automated system for Ki67 staining

IHC was performed using the fully automated Ventana Discovery XT (Roche Diagnostics, Rotkreuz, Switzerland). All steps were performed on the machine with Ventana solutions. Briefly, dewaxed and rehydrated paraffin sections were pretreated with heat using mild condition (20 min) CC1 solution. Sections were incubated with the primary antibody (1:100; Abcam, #ab16667) for 1 h at 37 °C. After incubation with OmniMap-anti-rabbit HRP (1:100), chromogenic revelation was performed with DAB (Roche). Sections were counterstained with haematoxylin.

### In situ hybridisation, RNA-ISH

In situ hybridisation for Fsp1 and Fak expression was performed on 5 μm FFPE sections using the RNAscope 2.5 HD Duplex (#322436) assay according to the manufacturers’ instructions (Advanced Cell Diagnostics, Newark, CA). RNAscope probes used were Mm-S100a4 (Fsp1, #412971), Hs-CD274 (FAK, #600861), Mm-Ccl6 (#510851), Mm-Ccl12 (#437521), Mm-Ppib (positive control probe, #313911) and 3-plex Negative Control Probe (#320871). Stained slides were stored at 4 °C before imaging on a Zeiss brightfield microscope.

### Pimonidazole detection of hypoxia

One hour prior to sacrifice, tumour-bearing mice were injected with 60 mg/kg pimonidazole hydrochloride (Hypoxyprobe™-1 HPI, Inc.) diluted in ddH_2_O to a final concentration of 10 mg/mL) intravenously via the tail vein. Tumours were snap frozen immediately after cervical dislocation. Five-micrometre cryosections were thawed, rehydrated and fixed for 10 min in −20 °C acetone. Sections were incubated with 1:50 anti-pimonidazole overnight at 4 °C. Sections were then washed and mounted with ProLong Gold™ with Antifade (Invitrogen). Images were taken with an Axioplan Zeiss microscope.

### Vascular perfusion

Vascular perfusion was visualised by injecting mice via the tail vein with 20 µg of PE-conjugated mouse monoclonal anti-PE-PECAM (Biolegend, #102408) 10 min prior to sacrifice. Tumours were snap frozen, sectioned and stained for endomucin as above. The percentage of double-positive blood vessels was presented as an indication of blood vessel perfusion.

### Western blotting

Cells were grown to 70–80% confluency. RIPA buffer was used for lysis for all experiments apart from those for PI3K inhibition where high SDS buffer was used instead. Five to 10 μg protein was run on 15% polyacrylamide gel for FSP-1 and 8–10% polyacrylamide gel for others and then transferred to nitrocellulose membranes. The membranes were blocked for 1 h in 5% milk in phosphate-buffered saline with 0.1% Tween-20 (PBS-T), followed by an overnight incubation of primary antibody diluted 1:1000 in 5 mL of 5% BSA in PBS-T at 4 °C. The blots were then washed three times with 10 mL of PBS-T and incubated with the relevant horseradish peroxidase (HRP)-conjugated secondary antibody diluted 1:2500 in 5% milk in PBS-T for 1 h at room temperature. After further washes in PBS-T, bands were detected by chemiluminescence. HSC70 or GAPDH were used as loading controls. The following antibodies were used: HSC70 (1:5000 dilution, Mouse monoclonal; Santa Cruz, #sc7298), GAPDH (1:5000 dilution, Mouse monoclonal, clone 6C5; Millipore, #MAB374) smooth muscle actin (1:1000 dilution, Mouse monoclonal, Clone 1A4; DAKO, #M0851), FAK (1:1000 dilution, Mouse monoclonal, Clone 4.47; Millipore, #05-537), FSP-1 (1:500 dilution, Rabbit polyclonal; Millipore, #07-2274), PDGFR-β (1;1000 dilution, Rabbit monoclonal, Clone 28E1; Cell Signaling, #3169), E-cadherin (1:1000 dilution, Rabbit monoclonal, Clone 24E10; Cell Signaling, #3195), Endomucin (1:1000 dilution, Rat monoclonal; Santa Cruz, #sc65495), Pyk2 (1:1000 dilution, Mouse monoclonal; Cell Signaling, clone 5E2, #3480), phospho-AKT (Ser473) (1:1000 dilution, Rabbit monoclonal, 193H12; Cell Signaling, #4058).

### Phenotypic analysis of tumours by flow cytometry

Tumours were minced and incubated at 37 °C for 20 min in an enzymatic cocktail containing DNase (0.5 mg/mL; Sigma). Collagenase type V (2 mg/mL; Sigma) in HBSS (Sigma) was used to make a single-cell suspension for pancreatic tumours. For breast tumours, collagenase/dispase (1 mg/mL, Sigma) in PBS was used to make a single-cell suspension.

Cells were passed through a 70 μM filter (BD Biosciences), washed in PBS supplemented with 2% foetal bovine serum and 2 mM EDTA, counted and used immediately for flow cytometry. Before cells were stained with specific antibodies, nonspecific binding sites were blocked with Fcγ R III/II TruStain fcX (93, Biolegend). Staining was performed in PBS supplemented with 2% foetal bovine serum and 2 mM EDTA.

The following fluorochrome-conjugated antibodies were used: anti-CD45 (1:100, 30-F11, #103149), anti-CD3ε (1:50, 145-2C11, #100320), anti-CD4 APC (1:200, RM4-4, #116014), anti-CD8APC/PE (1:200, 53-6.7, #100712, #100708), anti-CD69 (1:100, H1.2F3, #104506), anti-CD44 (1:100, IM7, #103049), anti-CD62L (1:100, MEL-14, #104438), anti-CD19PerCP (1:200, 6D5, #115532), anti-CD11b (1:100, M1/70, #101259), anti-F4/80 PE (1:50, BM8, #123110) all from Biolegend; anti-PD1 (1:100, RMP1-30, #48-9981-80), anti-Gr1 (1:200, RB6-8C5, #56-5931-82) and anti-Ly6C (1:100, HK1.4, 48-5932-82) all from eBioscience. Fixable viability dye (1:500, FVD, #65-0866-14) (eBioscience) was used to discriminate between live and dead cells. Acquisition and analyses were performed on a BD LSRII system using BD FACSDIVA software (BD Biosciences). The percentage of cells were analysed using FlowJo software (version 10.0.8 tree Star). Dead cells were excluded from the analysis on the basis of FVD and SSC gating. Cell doublets were excluded from the analysis by gating for FSC area versus FSC width. CD45+ cells were used to include leucocytes only in the data analysis. For Gating strategy please see Supplementary Fig. 8.

### Isolation and culture of bone marrow-derived macrophages

Tibias and femurs were harvested from mice. Under sterile conditions, the ends of the bone were held with sterile forceps and a 27G needle (BD Biosciences) syringe containing 10 mL of PBS was used to flush cells from the bone marrow. Single-cell suspensions were washed with PBS and resuspended in 5 mL of red blood cell lysis buffer (BD Biosciences).

Single-cell suspension was passed through a 70-μm cell strainer (BD Falcon) and counted. After centrifugation, cells were resuspended in complete medium and plated in a 150 mm bacterial Petri dish (Thermo Fisher Scientific) containing 20 ng/mL of recombinant CSF-1 (BD Biosciences) and incubated for 7 days at 37 °C in a 5 % v/v CO_2_ atmosphere.

### Magnetic resonance imaging

To monitor tumour growth, MR imaging using a Bruker ICON™ 1T MRI system (Bruker, Ettlingen, Germany) was performed on mice that had been subjected to orthotopic pancreas surgery. Mice were anaesthetised with 1.3–3% isoflurane at 1 L/min and maintained at 37 °C throughout the imaging procedure. Using a 30 mm i.d., ×50 mm mouse body coil, T2-weighted scans with respiratory gating were performed (acquisition software: ParaVision Acquisition 5.1) using the following acquisition protocol: pulse sequence (RARE), echo time (84.00 ms), repetition time (3149.139 ms), averages (4), rare factor (8), number of coronal slices (13), slice thickness (0.850 mm), slice gap (0.250 mm), matrix size (110 × 110), field of view (30 × 30mm), resolution (0.273 × 0.273 mm) and imaging time less than 3 min. Images were analysed using VivoQuant 3.0 analysis software (inviCRO LLC, Boston, MA). For quantitative analysis, total tumour volume was calculated by adding together the volumes (mm^3^) per slice of tumour for each individual mouse.

### ^18^F-FDG PET/CT Imaging

All animals were fasted overnight for at least 12 h prior to ^18^F-FDG PET/CT scans. Blood glucose was measured and the animals weighed prior to administration of 10–15 MBq of ^18^F-FDG in a volume of 200 µL via the tail vein. Animals were maintained under isoflurane anaesthesia (1.5% at 1 L/min) at 37 °C for a 60 min uptake period after which an attenuation CT scan was performed (10 min duration) followed by PET scanning under the same temperature and anaesthesia levels. A Siemens Inveon PET/CT scanner (Siemens Preclinical Solutions Knoxville, TN) was used in combination with Inveon Acquisition Workplace software (version 1.5 Siemens Medical Solutions MI) for all imaging procedures. PET data acquired using a 350–650 keV energy window were reconstructed to a 128 × 128 × 159 matrix with a voxel size of 0.776 mm × 0.776 mm × 0.796 mm using the 3D-ordered subsets expectation maximisation (OSEM3D) reconstruction algorithm with CT attenuation and scatter correction applied (2 OSEM3D iterations, 18 MAP Iterations). MicroCT scans were acquired using the factory set attenuation CT protocol with the following parameters: 3 bed positions (to match the PET field of view), 120 rotation steps over 220°, continuous rotation, 80 kVp tube voltage, 500 μA tube current, 200 ms exposure and a binning setting of 4 yielding an effective pixel size of 108.07 µm. The CT images were reconstructed using the factory set attenuation CT reconstruction protocol into a 384 × 384 × 604 matrix with an isotropic voxel size of 0.216 mm × 0.216 mm × 0.216 mm and Hounsfield calibration was applied. For quantitative assessment, images were analysed using VivoQuant 3.0 analysis software (inviCRO LLC, Boston, MA). PET and CT images were co-registered and volumes of interest (VOIs) were generated to match breast tumour volume. Uptake in the tumours was calculated as SUVmax.

### Infusions of ^13^C-labelled nutrients and dissection of tumours

For isotopomer analysis of glucose metabolism, tumour-bearing mice were injected with a bolus dose of 20 mg [U-^13^C6]-glucose (Cambridge Isotope Laboratories) via tail vein. Mice were sacrificed 15 min after the last injection. Tumours were dissected rapidly, snap frozen and stored at −80 °C.

### Sample preparation and LC-MS analysis

Snap-frozen tissue specimens were cut and weighed into Precellys tubes prefilled with ceramic beads (Stretton Scientific Ltd, Derbyshire, UK). An exact volume of extraction solution (30% acetonitrile, 50% methanol and 20% water) was added to obtain 40 mg specimen per mL of extraction solution. Tissue samples were lysed using a Precellys 24 homogeniser (Stretton Scientific Ltd, Derbyshire, UK). The suspension was mixed and incubated for 15 min at 4 °C in a Thermomixer (Eppendorf, Germany), followed by centrifugation (16,000 *g*, 15 min at 4 °C). The supernatant was collected and transferred into autosampler glass vials, which were stored at −80 °C until further analysis.

Samples were randomised in order to avoid bias due to machine drift and processed blindly. LC-MS analysis was performed using a QExactive Orbitrap mass spectrometer coupled to a Dionex U3000 UHPLC system (Thermo). The liquid chromatography system was fitted with a Sequant ZIC-pHILIC column (150 mm × 2.1 mm) and guard column (20 mm × 2.1 mm) from Merck Millipore (Germany) and temperature maintained at 45 °C. The mobile phase was composed of 20 mM ammonium carbonate and 0.1% ammonium hydroxide in water (solvent A), and acetonitrile (solvent B). The flow rate was set at 200 µL/min with the gradient described previously^63^. To expand on the range of metabolites covered in the analysis, the sample extracts were then run on a ZIC-HILIC column (150 mm × 4.6 mm) fitted with a guard column (20 mm × 2.1 mm) (both Merck Millipore, Germany). The aqueous mobile phase solvent used was 0.1% formic acid in water (solvent A) and the organic mobile phase was 0.1% formic acid in acetonitrile (solvent B). The flow rate was set at 300 μL/min and the column oven set to 30 °C. The mobile phase gradient was described previously^63^. The mass spectrometer was operated in full MS and polarity switching mode. The acquired spectra were analysed using XCalibur Qual Browser and XCalibur Quan Browser software (Thermo Scientific).

### Cytokine arrays

Wild type and FAK-depleted CAFs were grown in normal DMEM media supplemented with 10% FBS. Whole-cell lysates were extracted when cells were 70–80% confluent. Mouse XL cytokine arrays (Proteome Profiler ARY028, R&D Systems) were performed according to the manufacturer’s instructions using 100 μg of lysates per membrane. Pixel analysis was used for quantification with ImageJ software.

### Ccl6 and Ccl12 stimulation of primary MMTV-derived tumour cells

Tumour cells were starved for 12 h in serum-free culture medium followed by 5-h treatment with mouse recombinant Ccl6 (200 ng/mL; Peprotech, #250-06) and Ccl12 (5 ng/mL; BioLegend, #587904) before extracellular flux analysis. Concentration used are within physiological local tissue cytokine expression level ranges.

### Ccr1/Ccr2 siRNA of E0771 cells

E0771 cells were seeded either in a six-well plate (100,000 cells/well; for qRT-PCR) or into a Seahorse plate (30,000 cells/well), the day before transfection to achieve 60–70% confluency. For transfection of Ccr1 and Ccr2 siRNA (ON-TARGETplus from Horizon Discovery—12768, 12772), jetPRIME transfection reagent (Polyplus, #114-07) was used according to the manufacturer’s instructions. On-TARGETplus non-targeting pool (Horizon Discovery, #D-001810-10) was used as a negative control siRNA. Cells were incubated in their normal culture medium before and during transfection for 24 h. Ccr1, Ccr2 and non-targeting control siRNA were all used at 25 nM concentration. After 24 h, the medium was removed and replaced with either FAK-depleted CAF conditioned medium or FAK-WT-CAF conditioned medium for 4 h, or maintained in normal culture medium as a control. After 4 h, extracellular flux analysis experiments were performed. For the six-well plate, at the end of the 4 h incubation with conditioned medium, cells were pelleted and stored at −80 °C prior to RNA extraction. Quantitation of Ccr1 and Ccr2 levels: RNA was extracted from E0771 cells transfected with Ccr1 and Ccr2 siRNA using the RNA isolation kit according to the manufacturer’s instructions (RNeasy mini kit, Qiagen, cat no. 74106) and RNA concentrations were quantified using the Nanodrop spectrophotometer (Thermo Fisher Scientific). In all, 1 μg/μL RNA was converted to cDNA using the High Capacity cDNA reverse transcription kit (Thermo Fisher Scientific, #4368814). *Ccr1*, *Ccr2* and *Actin* primers and probes for qRT-PCR Taqman reactions were obtained from Applied Biosystems. cDNA samples were run in triplicate for each treatment. Gene expression levels of *Ccr1* and *Ccr2* were analysed using the StepOne Real Time PCR machine and software (Applied Biosystems).

### Quantitative real-time PCR

Primary MMTV-derived CAFs were lysed in RLT buffer (Qiagen). Total mRNA was isolated using the RNeasy Mini kit (Qiagen). Quality control and concentration of samples was carried out using a Nanodrop ND-10000 spectrophotometer. RNA was reverse transcribed using High Capacity cDNA Reverse Transcription kit (Applied Biosystems) according to the manufacturer instructions. Real-time PCR was performed in a StepOne Plus thermocycler (Applied Biosystems) using TaqMan Master mix and primers custom-made that were specific to mouse *Ccl6* (Mm01302419), mouse *Ccl12* (Mm01617100), mouse *Ccl7* (Mm00443113), mouse *Ccl8* (Mm01297183), mouse *Ccr2* (Mm01216173) and *Gapdh* (4352339E) all from Applied Biosystems. The data were normalised to *Gapdh* endogenous control to compensate for experimental variations. Fold changes were calculated using the comparative CT (cycle threshold) method.

### Seahorse XF^e^ Extracellular Flux Analyser experiments

For the assay, cells were plated in XFe96 Cell Culture Microplates (Agilent Technologies) at a cellular density of 30,000 cells/well as follows: EpCAM+ sorted malignant cells from fresh MMTV tumours were seeded on the day of the assay in previously-coated plates; E0771 cells with mock transfection, scr siRNA or Ccr1/Ccr2 siRNA transfection (see Ccr1/Ccr2 siRNA of E0771 cells); primary malignant cells or CAFs, that were in culture, were seeded 24 h prior to the assay. For oxygen consumption rate (OCR) determination, cells were incubated in base assay medium (Agilent Technologies) supplemented with 2 mM glutamine, 10 mM glucose and 1 mM pyruvate for 1 h, prior to the measurements using the XF Cell Mito Stress Kit (Agilent Technologies). Concentrations of oligomycin, FCCP, antimycin and rotenone were adjusted for each cell type. For glycolytic metabolism measurements, cells were incubated in basal media (Agilent Technologies) supplemented with 2 mM glutamine and 1 mM pyruvate for 1 h prior to injections using the Glycolysis Stress Test Kit (Agilent Technologies). Experiments were run in a XF96^e^ analyser (Agilent Technologies), and raw data were normalised to protein content calculated by Bradford method. Where appropriate, conditioned medium (CM) from WT and FAK-depleted CAFs were collected over a 48 h time period in normal culture medium. CM was either used fresh or boiled for 10 min to inactivate proteinaceous factors before applying to malignant cells for 2 or 48 h before flux analysis was performed.

### Fibroblast activation in vivo

Bleomycin A5 hydrochloride (#ab142406) was purchased from AbcamBiochemicals. Mice were anaesthetised using isoflurane and treated with 10 mg/kg (in saline) through intranasal instillation. Mice were culled 21 days after the treatment and fibroblasts were isolated.

### Drug treatments followed by qRT-PCR and extracellular flux analysis

For qRT-PCR, primary CAFs were treated with 1 µM PI3K inhibitor (GDC-0941; Selleckchem, #S1065) for 24 h or 5 µM FAK inhibitor (PF-573,228; Tocris, #3239) for 48 h. For seahorse experiments, cancer cells were exposed to conditioned medium from CAFs with 10 µM PKA inhibitor (KT 5720; Tocris, #1288), 10 µM PKC inhibitor (Go6983; Tocris, 2285/1#) for 3 h. Alternatively, cancer cells were incubated in CAF CM with 1 µM CCR1*i* (Chemocentryx) and 1 µM CCR2*i* (Chemocentryx) for 48 h.

### In vivo treatment of orthotopic breast tumour-bearing mice with CCR1*i*/CCR2*i*

CCR1*i* and CCR2*i* were discovered through structure–activity relationship modification of screening hits and synthesised by the Medicinal Chemistry Department at ChemoCentryx (Mountain View, CA) according to the procedures described (Patent Application Numbers: WO 2014089495 and WO 2016187393)^64^. HydroxylPropyl MethylCellulose (HPMC, The Dow Chemical Company, Midland, MI, USA) was employed as the suspending agent in formulations for in vivo experiments. HPMC was dissolved in USP Sterile Water (Mediatech, Inc., Manassas, VA, USA) and the placebo vehicle was 1% w/v HPMC. Drug formulations were prepared by weighing the required amount of CCR1*i* and CCR2*i* into a mortar, followed by slow addition of 1% w/v HPMC vehicle into the mortar with continuous mixing with a pestle until a homogeneous suspension was produced. The concentrations of CCR1*i* and CCR2*i* in the formulation were both 6 mg/mL (30 mg/Kg dose and 5 mL/Kg dosing volume). The inhibition of chemotaxis was assessed in WEHI-274 murine monocyte cell line^64^ that endogenously express CCR1 and CCR2. Employing the WEHI-274 cell line, independently CCR1*i* and CCR2*i* inhibited CCR1 and CCR2 mediated chemotaxis with IC_50_ of 20 nM, respectively, in 100% mouse serum.

*FSP-Cre*+*;FAK*^*fl/fl*^ mice were dosed by oral gavage with CCR1*i* and CCR2*i* once daily (1% HPMC in water) both at 30 mg/kg. *FSP-Cre−;FAK*^*fl/fl*^ mice were dosed 1% HPMC in water in parallel as the vehicle control. Dosing began on day 8 and continued until day 20 for LC-MS or day 28 for tumour growth experiments. Trough plasma levels were above the IC_90_ for each compound resulting in complete CCR1 and CCR2 receptor engagement by CCR1*i* and CCR2*i*, respectively.

### CCR1i and CCR2i specificity assays

The following describes each assay in the following format: receptor: cell type/ligand/function tested. CCR1: THP-1 cells/lymphotactin/cell migration; CCR2: peripheral blood mononuclear cells/MCP1/Ca2+ flux; CCR3: transfected 293 cells/eotaxin/Ca2+ flux. CCR4: peripheral blood T cells/MDC/Ca2+ flux; CCR5: transfected L1/2 cells/labelled MIP-1β/ligand binding; CCR6: peripheral blood T cells/MIP-3α/Ca2+ flux; CCR7: peripheral blood T cells/MIB-3β/Ca2+ flux; CCR8: transfected 293 cells/I309/Ca2+ flux; CCR9: MOLT-4 cells/TECK/cell migration; CCR10: transfected 293 cells/CCL28/Ca2+ flux; CXCR1: peripheral blood neutrophils/IL8/Ca2+ flux; CXCR2: peripheral blood neutrophils/GRO-α/Ca2+ flux; CXCR3: peripheral blood lymphocytes/ITAC/Ca2+ flux; CXCR4: peripheral blood lymphocytes/SDF1α/Ca2+ flux; CXCR5: transfected L1/2 cells/BCA-1/Ca2+ flux; CXCR6: peripheral blood lymphocytes/CXCL16/Ca2+ flux; C3aR: /peripheral blood neutrophils/complement C3a/Ca2+ flux; C5aR/peripheral blood neutrophils/complement C5a/Ca2+ flux; FPRL1: peripheral blood neutrophils/CCL23(aa_22–137_)/Ca2+ flux.

### Gene expression data analysis and clinical inferences

Published datasets for breast cancer^16^ and pancreatic cancer^18^ were used for the human cancer stroma gene expression analysis. Gene expression profiling (GEP) data of tumour-associated stroma derived from primary breast cancer samples (*n* = 53)^16^ were extracted and used to inspect the association between FAK expression in stroma and overall survival (OS). Of the three probes mapped to FAK/PTK2 gene, the two probes (Agilent-012391 Whole Human Genome Oligo Microarray G4112A, platform GPL1708, probe 1: Agilent feature number 4302; probe 2: Agilent feature number 11888) with the most expression abundance were used for the following analysis (Extended Fig. 1a). The high- and low-expression groups were determined using the method described previously^65^. Briefly, each percentile of expression between lower and upper quartiles was used in the Cox proportional hazards (Coxph) regression analysis and the best performing threshold of percentile associated with OS was determined. Survival modelling and Kaplan–Meier (KM) analysis was undertaken using R “survival” package. OS was defined as time from diagnosis to death or to the last follow-up date for survivors. We further assessed the clinical association of FAK expression using the multivariate analysis, accounting for age, tumour grade, ER status, tumour size, status of radiotherapy, hormone-therapy and chemotherapy. Hazard ratio (HR) and 95% confidence interval (CI), as well as associated *P* values at the best performing threshold, were then derived. The KM analysis was also performed using the median cutoff (equal number split), and the similar trend was observed to that using the best performing threshold.

A primary pancreatic cancer dataset^18^ was also selected, with both GEP and clinical data available (*n* = 102), for the clinical inference of FAK expression. Samples enriched for “activated” stroma genes were further identified (*n* = 54) based on the stroma signature derived from Moffitt et al.^17^ using the non-negative matrix factorisation (NMF) consensus clustering^66^ (Extended Fig. 1d). Gene signature for activated stroma seemed to be associated with an activated fibroblast state. Within this set of activated stroma samples, the association between FAK expression and OS was assessed using the procedure described above, accounting for tumour stage (the only available clinical parameter in addition to OS).

### Differential analysis of epithelial gene expression

For the GEP data of tumour-associated stroma, matched LCM epithelial samples and their GEP data were available for 9 out of 10 low and 39 out of the 43 high stromal FAK samples (Finak et al. dataset). This allowed us to perform the differential expression (DE) analysis in the LCM epithelial compartment between low and high stroma FAK groups, to determine the association between stroma FAK and epithelial gene expression patterns. DE analysis in epithelial gene expression was performed using limma^67^, and significantly differentially expressed probes/genes were identified using false discovery rate (FDR) <0.05. The gene expression values, presented as the normalised log-ratio of test RNA over reference RNA, for our top candidate genes were also shown as box plots between low and high stromal FAK groups. The reference RNA used in Finak et al. dataset was Universal Human Reference RNA (Stratagene, ID #740000, La Jolla, California, USA). For the comparison of individual targets between low and high stromal FAK patients, two-tailed Student’s *t*-test was used.

### Pathway analysis

DE statistics derived from the limma test of epithelial gene expression were further ranked based on log2 fold changes of low versus high stroma FAK groups, and were used as input for the gene-set enrichment analysis (GSEA) to identify dysregulated pathway gene sets curated in the Molecular Signatures Database (v6.0)^68^. KEGG and Reactome gene sets were selected. Significantly dysregulated pathways were identified (FDR < 0.1), and the network of selected significant pathways was further constructed using the Cytoscape network visualisation software, Enrichment Map^69^.

### Proteomics and phosphoproteomics

For proteomics and phosphoproteomics studies, cell lysis and trypsin digestion was performed as previously described^70^. Phosphopeptides were enriched using TiO_2_ (GL Sciences)^70^. For phosphoproteomics, dried peptide pellets were resuspended in 9 µL of reconstitution buffer (20 fmol/µL enolase digest in 3% ACN, 0.1% TFA) and 5.0 µL were loaded onto an LC-MS/MS system consisting of a Dionex UltiMate 3000 RSLC directly coupled to an Orbitrap Q-Exactive Plus mass spectrometer (Thermo Fisher Scientific). For proteomics, pellets were resuspended in reconstitution buffer (0.5 µg/µL) and 2 µL were injected. The LC system used mobile phases A (3% ACN: 0.1% FA) and B (100% ACN; 0.1% FA). Peptides were trapped in a μ-pre-column and separated in a nanoflow analytical column. The following parameters were used: 3–28% B gradient for 120 min and a flow rate of 0.25 µL/min. Eluting peptides were analysed in a Q-Exactive Plus system with scan survey spectra (*m*/*z* 375–1500) was followed by, data-dependent acquisition of the 15 most intense ions were selected for HCD (higher energy collisional dissociation) and MS/MS scanning (200–2000*m*/*z*) with a resolution of 17,500 FWHM. A 30 s dynamic exclusion period was enabled with 10 ppm mass window.

Peptide identification and quantification was carried out from the MS/MS and MS data using the Mascot search engine as described before^71^. KSEA was carried out by grouping peptides into substrate sets known to be phosphorylated by a specific kinase^38^. Gene ontology enrichment analysis of proteins differentially phosphorylated between conditions (at *P* < 0.05) was carried out using the hypergeometric test^72^. For gene ontology analysis, we selected phosphopeptides with *P* < 0.05. For KSEA we do not have a threshold for selection as the enrichment is calculated using all detected phosphopeptides. The mass spectrometry proteomics data have been deposited to the ProteomeXchange Consortium via the PRIDE partner repository with the dataset identifier PXD008276 and 10.6019/PXD008276.

### Statistical analysis

Results are presented as means ± s.e.m. for at least 2–3 independent experiments, unless otherwise stated. The sample sizes used were based on level of changes and consistency expected. Statistical significance was reported as appropriate. For animal experiments, animals were excluded from the analysis if tumour volume breached the Home Office legal size limit. During animal experiments, the investigator was blinded to the genotype of the animals under study. For the tumour growth experiments, two-way ANOVA was used for statistical analysis. For the remaining experiments, *P* values were calculated with the two-tailed unpaired Student’s *t*-test unless otherwise stated. *P* < 0.05 was considered statistically significant.

### Ethical regulations

All procedures were approved by our local animal ethics committee, Queen Mary University of London, and were executed in accordance with United Kingdom Home Office regulations.

### Reporting summary

Further information on research design is available in the Nature Research Reporting Summary linked to this article.

## Supplementary information

Supplementary Information

Reporting Summary

## Data Availability

For gene expression profiling (GEP), publicly available datasets were obtained and used, GSE9014 and GSE2150. The mass spectrometry proteomics data have been deposited to the ProteomeXchange Consortium via the PRIDE partner repository with the dataset identifier PXD008276 and 10.6019/PXD008276 (https://www.ebi.ac.uk/pride/archive/projects/PXD008276). All the relevant data that support the findings of this study are available from the corresponding author on request.
