# Supplementary material for: Cancer associated fibroblast FAK regulates malignant cell metabolism
Source: Nat Commun. 2020 Mar 10;11:1290. doi: 10.1038/s41467-020-15104-3 (PMC7064590; doi:10.1038/s41467-020-15104-3)
Supplement: Supplementary file 1 — Supplementary Information [file 41467_2020_15104_MOESM1_ESM.pdf]

Cancer associated fibroblast FAK regulates malignant cell metabolism

Demircioglu *et al*.

Supplementary Figure 1, Demircioglu *et al.*

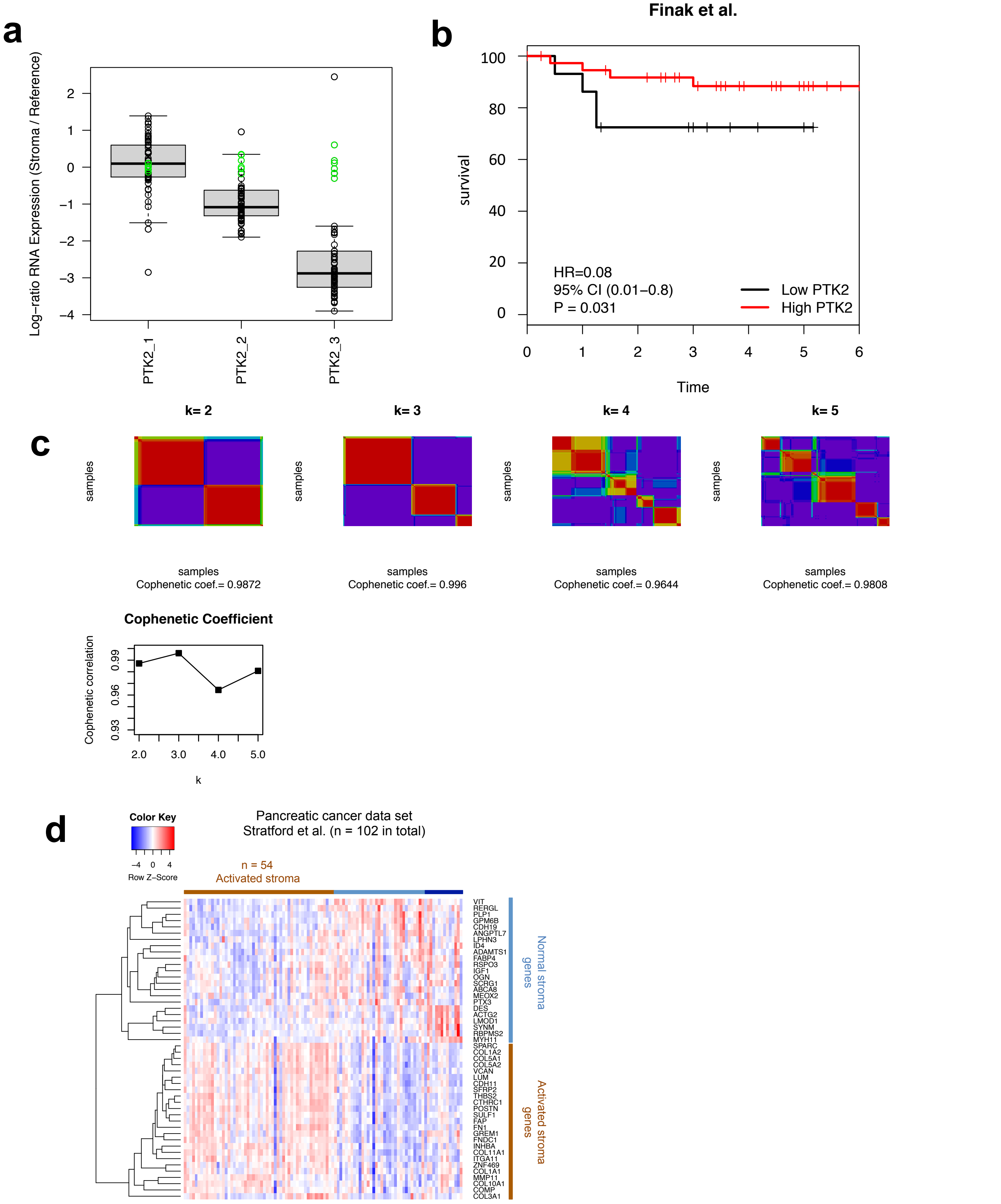

Supplementary Figure 1. Probe and patient selection rationale for multivariate analysis in human breast and pancreatic cancer patient datasets.

(a) Expression levels of three probes for stromal FAK (PTK2). Expression for tumour stroma and normal stroma were indicated by black and green circles, respectively. Probe 3 (Agilent-012391 Whole Human Genome Oligo Microarray G4112A, platform GPL1708. Agilent feature number, 30462) had the lowest expression levels in tumour stroma, thus was excluded from our analysis. For main Figure 1a, we show data using Probe 1 (PTK2_1, Agilent feature number, 4302). N=59, including stromal samples derived from 53 primary breast tumours (black circles) and 6 morphologically normal stroma (green circles). The box and whisker plot - box denotes the interquartile range (IQR, Q1 25th percentile – Q3 75th percentile), and whisker denotes the maximum (Q3 + 1.5*IQR) and minimum (Q1 – 1.5*IQR). **(b)** KM plot using PTK2 probe 2 (Agilent feature number, 11888) with overall survival (OS). For survival analysis, the Cox proportional hazards (Coxph) regression analysis was performed, and the log rank p-value was shown. (**c, d)** Identification of tumour samples with activated stroma in Stratford et al. pancreatic cancer patient data set (n=102). **(c)** In order to select the patients with activated stromal signatures, non-negative matrix factorisation (NMF) was performed based on the stroma signature from Moffitt *et al*., and cluster number k=3 provided the best clustering solution according to the Cophenetic Coefficient score. **(d)** Expression heatmap of stroma signature across all 102 pancreatic cancer samples. Samples with activated stroma (n=54) were identified with the high level of expression for activated stroma genes, and highlighted.

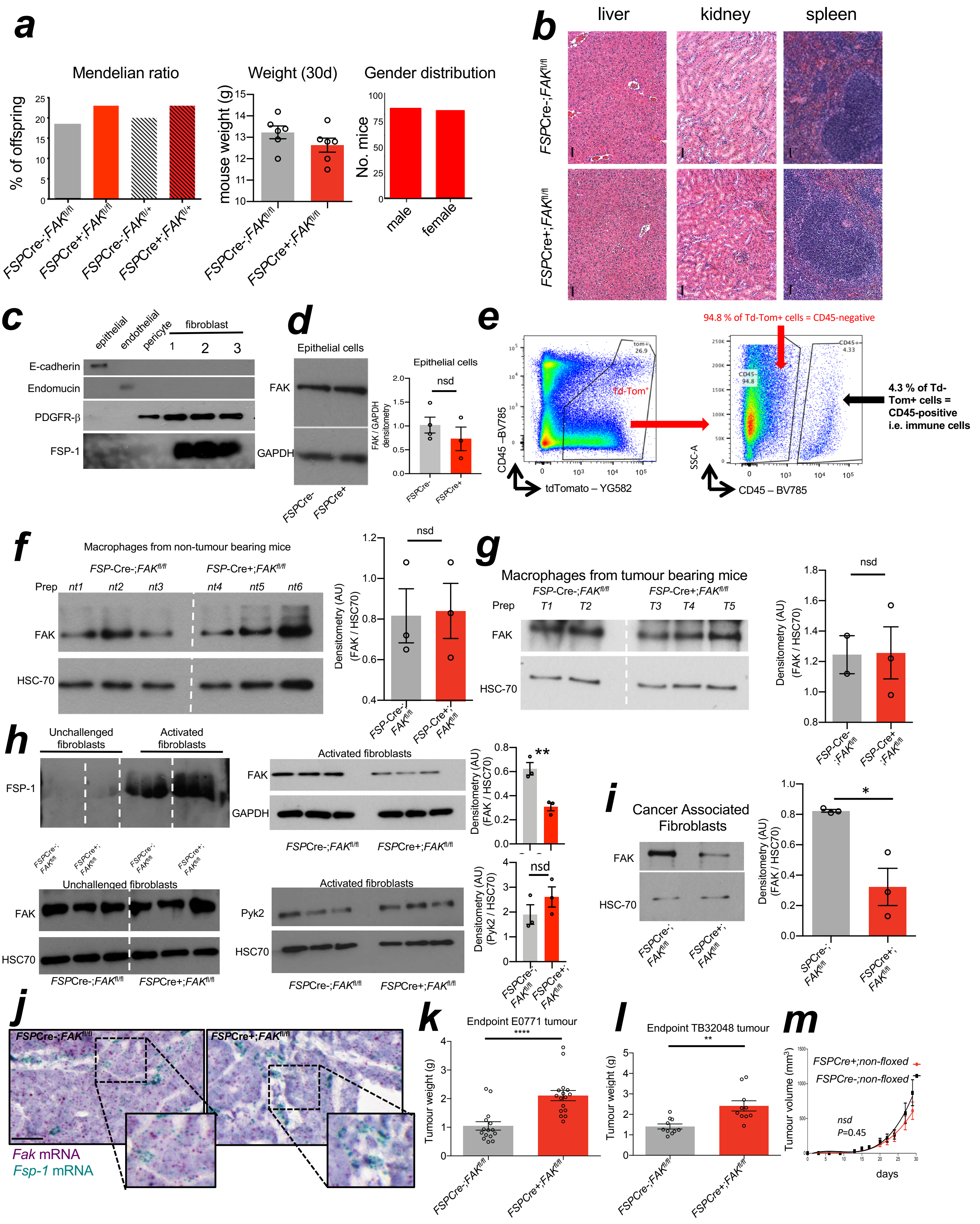

a
Mendelian ratio
% of offspring
FSPCre-;FAK fl/fl
FSPCre+;FAK fl/fl
FSPCre-;FAK fl/+
FSPCre+;FAK fl/+
Weight (30d)
mouse weight (g)
Gender distribution
No. mice
male
female
b
liver
kidney
spleen
c
epithelial
endothelial
pericyte
fibroblast
E-cadherin
Endomucin
PDGFR-β
FSP-1
d
Epithelial cells
FAK
GAPDH
FSPCre-
FSPCre+
nsd
FAK / GAPDH densitometry
e
94.8 % of Td-Tom+ cells = CD45-negative
Td-Tom+
CD45 –BV785
tdTomato – YG582
SSC-A
CD45 – BV785
4.3 % of Td-Tom+ cells = CD45-positive i.e. immune cells
f
Macrophages from non-tumour bearing mice
FSP-Cre-;FAK fl/fl
FSP-Cre+;FAK fl/fl
Prep
nt1
nt2
nt3
nt4
nt5
nt6
FAK
HSC-70
Densitometry (AU) (FAK / HSC70)
g
Macrophages from tumour bearing mice
T1
T2
T3
T4
T5
h
Unchallenged fibroblasts
Activated fibroblasts
FSP-1
FAK
GAPDH
Pyk2
HSC70
Densitometry (AU) (Pyk2 / HSC70)
i
Cancer Associated Fibroblasts
j
Fak mRNA
Fsp-1 mRNA
k
Endpoint E0771 tumour
****
Tumour weight (g)
l
Endpoint TB32048 tumour
**
m
FSPCre+;non-floxed
FSPCre-;non-floxed
nsd
P=0.45
Tumour volume (mm3)
days

Supplementary Figure 2. Characterisation of the *FSP-Cre+;FAK$^{fl/fl}$* mice shows no apparent gross defects but depletion of FAK in CAFs.

(a) Offspring genotypes after breeding *FSP-Cre+;FAK$^{fl/+}$* males with *FSP-Cre-;FAK$^{fl/fl}$* females follow normal Mendelian ratios. Bar charts show percentage of offspring genotypes. n = 37 *FSP-Cre-;FAK$^{fl/fl}$* (21.9%), 46 *FSP-Cre+;FAK$^{fl/fl}$* (27.2%), 40 *FSP-Cre-;FAK$^{fl/+}$* (23.7%), 46 *FSP-Cre+;FAK$^{fl/+}$* (27.2%). Body weights of the 30-day old female *FSP-Cre-;FAK$^{fl/fl}$* and *FSP-Cre+;FAK$^{fl/fl}$* mice. Bar chart shows mean mouse weight (g) ± s.e.m. n = 6 *FSP-Cre-;FAK$^{fl/fl}$* and 6 *FSP-Cre+;FAK*$^{fl/fl}$ mice. Gender distribution of the offspring after breeding *FSP-Cre+;FAK$^{fl/fl}$* males with *FSP-Cre-;FAK$^{fl/fl}$* females is normal. Bar chart shows the number of mice. n = 85 males and 84 females. **(b)** H&E stained sections of spleen, kidney and liver from non-tumour burdened *FSP-Cre+;FAK$^{fl/fl}$* and *FSP-Cre-;FAK$^{fl/fl}$* mice showed no gross differences in basic morphology. N=3 slides/organ/genotype. **(c)** Western blot analysis to assess the purity of primary fibroblast cultures. TB32048 pancreatic cancer cell line, primary lung endothelial cells and primary brain pericytes are used as controls. E-cadherin and endomucin were not expressed in fibroblast preps whilst they were positive for the tested markers of fibroblasts PDGFR-β and FSP-1. N=1 TB32048, lung endothelial cell and brain pericyte prep and n=3 fibroblast preps from 3 mice. **(d)** Western blot analysis of epithelial cell lysates from breast tumours grown in *MMTV+;FSP-Cre+; FAK$^{fl/fl}$* and *MMTV+;FSP-Cre-; FAK$^{fl/fl}$* mice show no change in FAK expression; n=4 *MMTV+;FSP-Cre-; FAK$^{fl/fl}$* cell preps, n=3 *MMTV+;FSP-Cre+; FAK$^{fl/fl}$* cell preps. Bar chart shows mean densitometric readings ± s.e.m. Two-sided Student's t-test. **(e)** Flow cytometric analysis of *tdTomato; MMTV+;FSP-Cre+* tumours indicate that 94.8% of tdTomato+ cells are CD45- (ie fibroblasts) whilst 4.3% of tdTomato+ cells are CD45+. **(f, g)** Western blot analysis of macrophages from either non-tumour bearing (nt) *FSP-Cre+; FAK$^{fl/fl}$* and *FSP-Cre-; FAK$^{fl/fl}$* or tumour bearing (T) *MMTV+;FSP-Cre+; FAK$^{fl/fl}$* and *MMTV+;FSP-Cre-; FAK$^{fl/fl}$* mice show no change in FAK expression. HSC-70 acts as a loading control. Bar chart represents densitometric reading, means + s.e.m. n=3 independent preparations of macrophages. **(h)** *FSP-Cre+;FAK$^{fl/fl}$* and *FSP-Cre-;FAK$^{fl/fl}$* mice were treated with bleomycin to induce lung fibroblast activation *in vivo* followed by primary fibroblast isolation. Western blot analysis revealed elevated FSP-1 expression in both genotypes after activation in vivo. Activated lung fibroblasts showed reduced expression of FAK when cells were isolated from *FSP-Cre+;FAK$^{fl/fl}$* mice. Expression levels of Pyk2 in activated fibroblasts were not significantly different between genotypes. Bar charts show mean densitometry (AU) ± s.e.m. n = 3 fibroblast preparations per genotype. HSC70 was used as a loading control.

(i) Western blot analysis of CAF lysates isolated from breast tumours in *MMTV+;FSP-Cre+;FAK*$^{fl/fl}$ and *MMTV+;FSP-Cre-;FAK*$^{fl/fl}$ mice show a significant depletion of FAK in CAFs isolated from *MMTV+;FSP-Cre+;FAK*$^{fl/fl}$ mice. HSC-70 acts as a loading control. Bar chart represents densitometric readings, mean + s.e.m., n= 3 independent replicates. **(j)** RNAscope *in situ* hybridisation was performed on tumour sections to assess reduction of FAK expression levels in FSP-1 positive CAFs *in vivo*. The number of purple dots representing FAK RNA transcripts was reduced in the cells which expressed green dots, representing FSP-1 RNA transcripts, in tumours from *MMTV+;FSP-Cre+; FAK*$^{fl/fl}$ mice but not from *MMTV+; FSP-Cre-;FAK*$^{fl/fl}$ mice. Scale bars (**b**) 50µm, (**j**) 50µm. **(k, l)** Late-stage tumour weights of **(k)** E0771 orthotopic breast tumours and, **(l)** TB32048 orthotopic tumours grown in *FSP-Cre+;FAK*$^{fl/fl}$ and *FSP-Cre-;FAK*$^{fl/fl}$ mice. Tumours grown in *FSP-Cre+;FAK*$^{fl/fl}$ mice were significantly heavier compared to control tumours. Bar charts show mean tumour weight ± s.e.m. n=15 E0771 and 10 TB32048 *FSP-Cre-;FAK*$^{fl/fl}$ tumours and 16 E0771 and 10 TB32048 tumours from *FSP-Cre+;FAK*$^{fl/fl}$ mice. **(m)** Orthotopic breast tumour growth was similar in *FSP-Cre+;non-floxed* and *FSP-Cre-;non-floxed* mice thus excluding the potential effect of Cre expression alone on tumour growth. Graph shows mean tumour volumes ± (s.e.m). n = 7 *FSP-Cre+*;non-floxed and 7 *FSP-Cre-;*non-floxed mice tumours. *P<0.05, ***P*<0.01, *****P*<0.0001, nsd, no significant difference. Statistical analysis (d, f, g, h, i, k, l) two-sided Student's t-test and (m) Two-way ANOVA.

Supplementary Figure 3, Demircioglu et al.

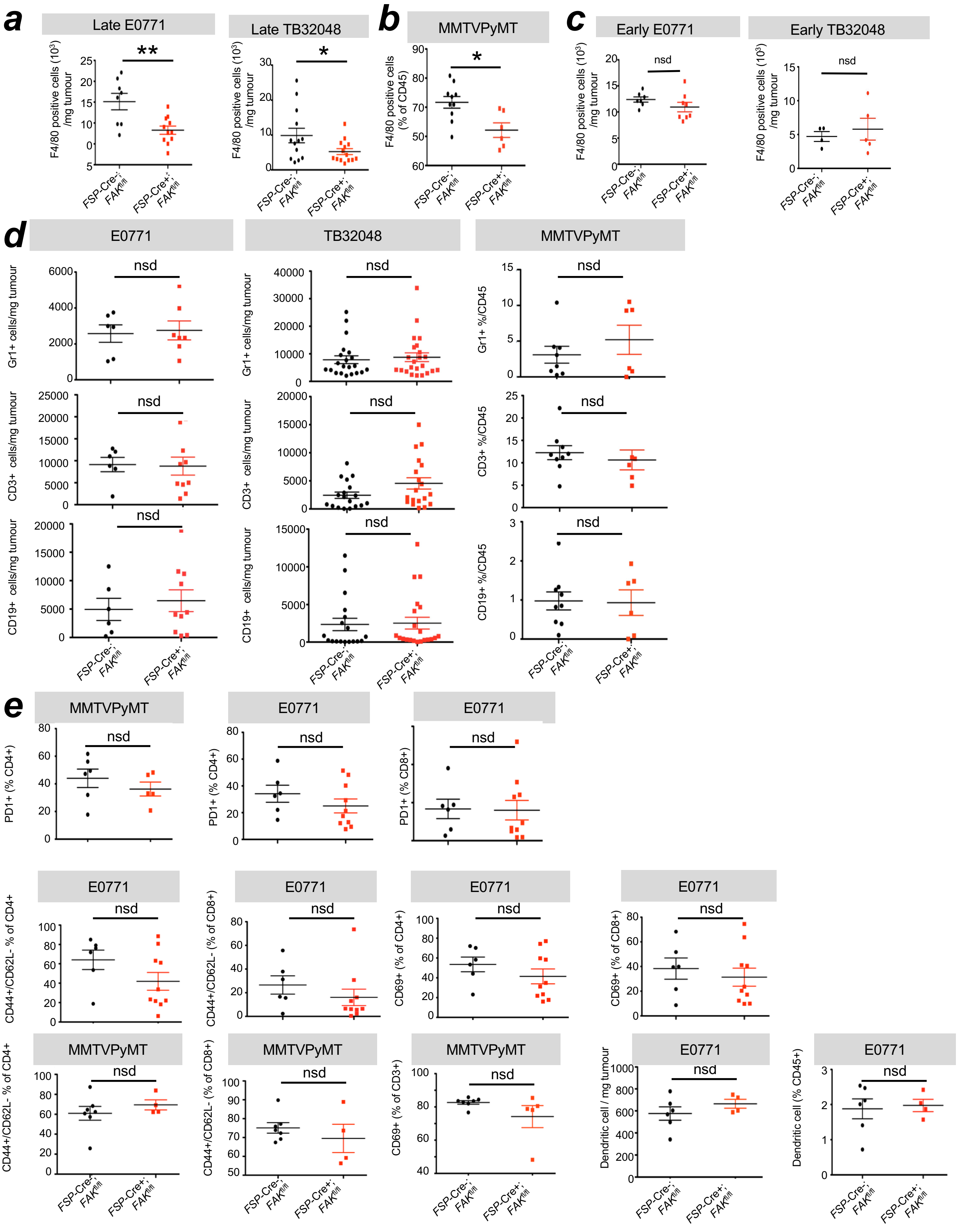

Supplementary Figure 3. ***FSP-Cre+; FAK$^{fl/fl}$*** **mice show reduced TAMs, but no change in the numbers and activation of T-lymphocytes as well as in the numbers of granulocytes, B-lymphocytes and dendritic cells.**

Flow cytometry analysis of fresh whole tumour cell suspensions showed that tumour associated macrophages (F4/80+) are reduced in: **(a)** late-stage orthotopic breast (E0771; n=8 *FSP-Cre-; FAK$^{fl/fl}$* and 11 *FSP-Cre+; FAK$^{fl/fl}$*) and pancreatic (TB32047; n=13 *FSP-Cre-; FAK$^{fl/fl}$* and 14 *FSP-Cre+; FAK$^{fl/fl}$*) tumours grown in *FSP-Cre+; FAK$^{fl/fl}$* mice and **(b)** spontaneous *MMTV+; FSP-Cre+; FAK$^{fl/fl}$* (n=10 *FSP-Cre-; FAK$^{fl/fl}$* and 6*FSP-Cre+; FAK$^{fl/fl}$* tumours) but not **(c)** early-stage tumours (E0771; n=7 *FSP-Cre-; FAK$^{fl/fl}$ and 8 FSP-Cre+; FAK$^{fl/fl}$* tumours. TB32048: n=4 *FSP-Cre-; FAK$^{fl/fl}$* and 5 *FSP-Cre+; FAK$^{fl/fl}$* tumours). Scatter plots show F4/80 +TAMs / mg (tumour weight) and % of F4/80+ TAMs/CD45+ immune cells in MMTV model mean± s.e.m. **(d)** Gr1+, CD3+ and CD19+ cell numbers are similar in E0771 (n=6 *FSP-Cre-; FAK*$^{fl/fl}$ and 7-10 *FSP-Cre+; FAK$^{fl/fl}$* tumours), TB32048 (n=18-21 *FSP-Cre-; FAK*$^{fl/fl}$ and 21-23 *FSP-Cre+; FAK*$^{fl/fl}$) and MMTVPyMT (n=8-9 *FSP-Cre-; FAK*$^{fl/fl}$ and 5-6 *FSP-Cre+; FAK*$^{fl/fl}$) tumours grown in *FSP-Cre+; FAK$^{fl/fl}$* and *FSP-Cre-; FAK$^{fl/fl}$* mice. **(e)** Numbers of activated T-lymphocytes are unchanged in tumours grown in *FSP-Cre+; FAK$^{fl/fl}$* and *FSP-Cre-; FAK$^{fl/fl}$* mice. nsd, no significant difference. Scatter plots represent mean ± s.e.m. n= 6-7 *FSP-Cre-; FAK$^{fl/fl}$* and 4-5 *FSP-Cr+-; FAK$^{fl/fl}$* MMTVPyMT tumours; n=6 *FSP-Cre-; FAK$^{fl/fl}$* and 4-10 *FSP-Cr+-; FAK$^{fl/fl}$* E0771tumours. *P<0.05, **P<0.01, nsd, no significant difference. Statistical analysis, two-sided Student's t-test.

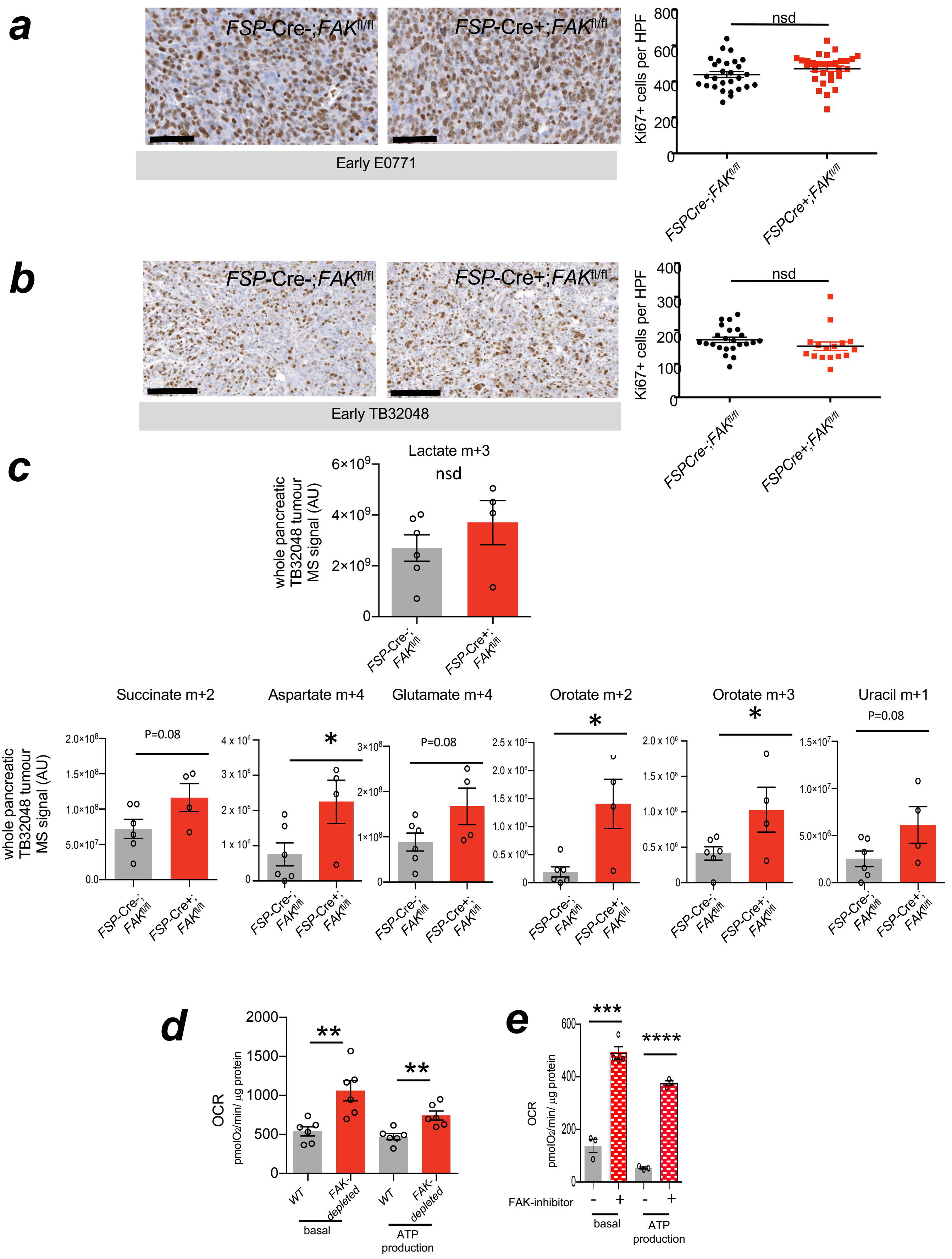
a
FSP-Cre-;FAKfl/fl
FSP-Cre+;FAKfl/fl
Early E0771
Ki67+ cells per HPF
nsd
FSPCre-;FAKfl/fl
FSPCre+;FAKfl/fl
b
FSP-Cre-;FAKfl/fl
FSP-Cre+;FAKfl/fl
Early TB32048
Ki67+ cells per HPF
nsd
FSPCre-;FAKfl/fl
FSPCre+;FAKfl/fl
c
Lactate m+3
nsd
whole pancreatic TB32048 tumour MS signal (AU)
FSP-Cre-; FAKfl/fl
FSP-Cre+; FAKfl/fl
Succinate m+2
P=0.08
Aspartate m+4
*
Glutamate m+4
P=0.08
Orotate m+2
*
Orotate m+3
*
Uracil m+1
P=0.08
d
OCR
pmolO2/min/ µg protein
**
**
WT
FAK-depleted
basal
ATP production
e
OCR
pmolO2/min/ µg protein
***
****
FAK-inhibitor
basal
ATP production

Supplementary Figure 4. Cell proliferation is unchanged in size-matched, early-stage tumours grown in *FSP-Cre-;FAK$^{fl/fl}$* and *FSP-Cre+;FAK$^{fl/fl}$* mice and LC-MS analysis of pancreatic tumours.

(a, b) Before tumour growth diverged significantly between genotypes (day 21 post-injection), early-stage, size-matched tumours grown in *FSP-Cre-;FAK$^{fl/fl}$* and *FSP-Cre+;FAK$^{fl/fl}$* mice were immunostained for Ki67 to compare cell proliferation between the two genotypes. **(a, b)** Representative histological images and quantification of Ki67 staining in **(a)** breast E0771 and **(b)** pancreatic TB32048 tumours. Scatter plots represent mean number of Ki67+ cells/per field ± s.e.m. n= 23 TB32048 and 28 E0771 *FSP-Cre-;FAK$^{fl/fl}$* and n=16 TB32048 and 30 E0771 *FSP-Cre+;FAK$^{fl/fl}$* high power fields (HPF) from n=3 (TB32048 and E0771 *FSP-Cre-;FAK$^{fl/fl}$*) and 4 (TB32048 and E0771 *FSP-Cre+;FAK$^{fl/fl}$*) tumours. Scale bar in **a** and **b**, 100 mm. **(c)** *FSP-Cre+;FAK$^{fl/fl}$* (n=4) and *FSP-Cre-;*FAK$^{fl/fl}$ (n=6) mice bearing early-stage, size-matched TB32048 tumours were infused with [U-^{13}C] glucose for 15 minutes and LC-MS analysis of metabolites, extracted from frozen tumours, was carried out. Bar charts show mean peak area (AU) ± s.e.m. **(d)** Extracellular flux analysis (Mito Stress test) of primary FAK-depleted and WT-CAFs. Basal respiration and ATP production are significantly increased in FAK-depleted CAFs; Bar chart shows mean OCR ± s.e.m. n=4 independent experiments, 6 technical repeats for each genotype from the representative run. **(e)** OCR from extracellular flux analysis of WT-CAFs treated with 5 μM FAK kinase inhibitor (PF-573,228) or DMSO. Bar chart shows mean OCR ± s.e.m. n=2 independent experiments, 3 WT-CAF – inhibitor and 4 WT-CAFS + inhibitor technical repeats for each treatment arm from separate primary CAF cell preparation. **P*<0.05, nsd, no significant difference. Statistical analysis, two-sided Student's t-test.

a

NAME	FDR q-val
KEGG_CITRATE_CYCLE_TCA_CYCLE	0.006614237
KEGG_PYRIMIDINE_METABOLISM	0.010517598
KEGG_RNA_POLYMERASE	0.0124988
KEGG_PURINE_METABOLISM	0.043714773
KEGG_ALANINE_ASPARTATE_AND_GLUTAMATE_METABOLISM	0.05413519
KEGG_FRUCTOSE_AND_MANNOSE_METABOLISM	0.051361676
KEGG_PROTEIN_EXPORT	0.06236223
KEGG_CELL_CYCLE	0.073049456
KEGG_ARGININE_AND_PROLINE_METABOLISM	0.071276024
KEGG_GLUTATHIONE_METABOLISM	0.07343983
KEGG_PPAR_SIGNALING_PATHWAY	0.071564734
KEGG_RIBOSOME	0.07427067

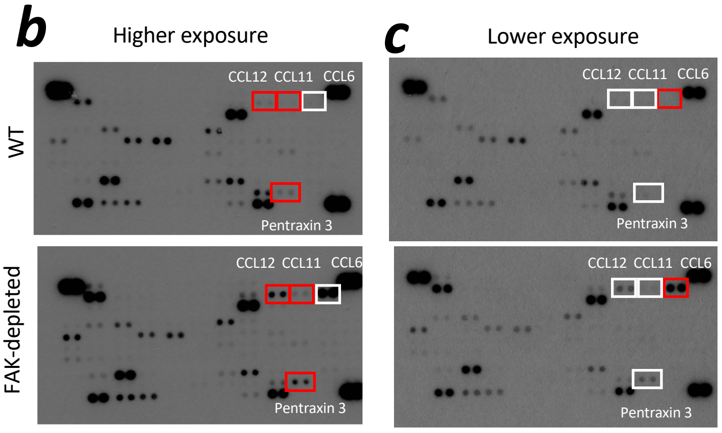

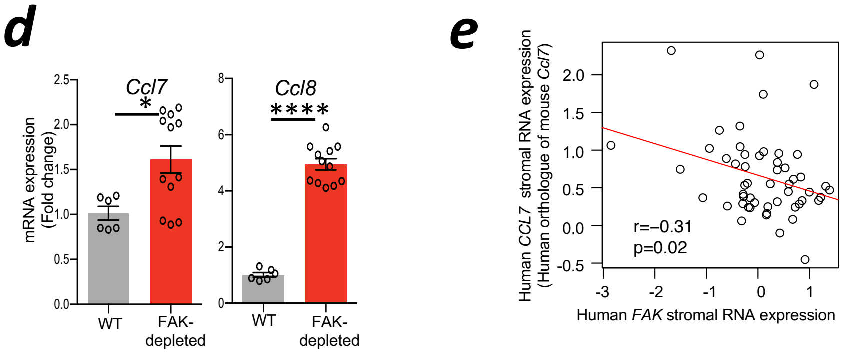

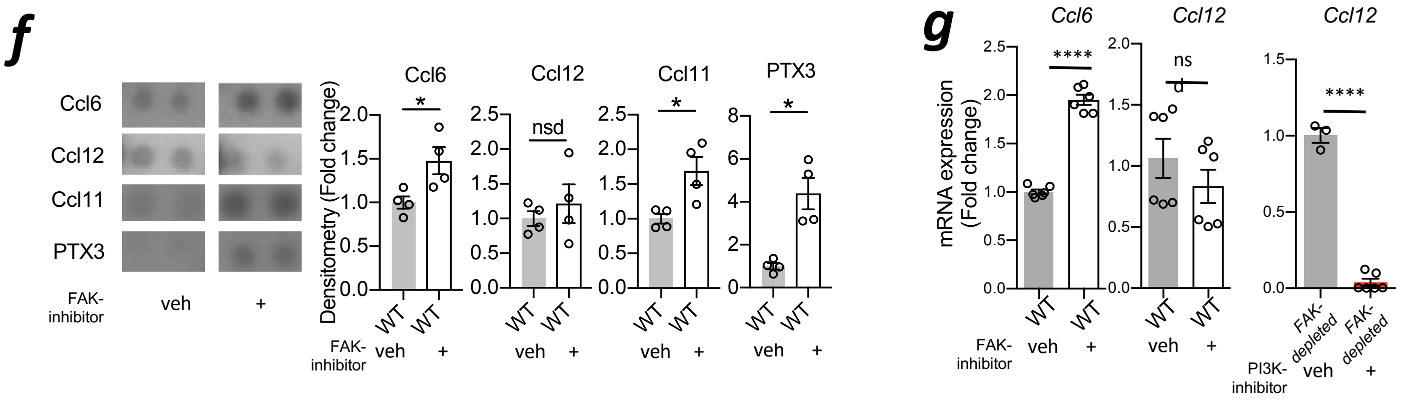

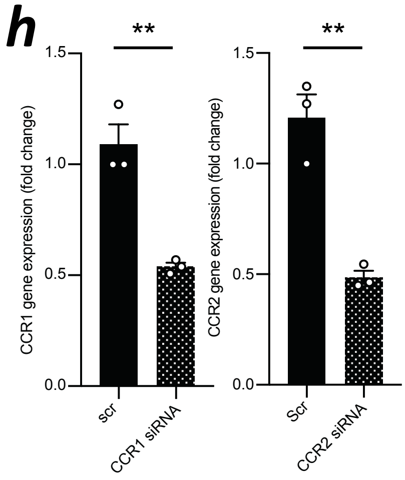

i

Ctnna1(S641);	**	Catenin alpha-1
Ctnnb1(S552);	**	Catenin beta-1
Itpr3(S1832);	**	Inositol 1,4,5-trisphosphate receptor type 3
Camkk2(S99);	**	Calcium/calmodulin-dependent protein kinase kinase 2
Camkk2(S511);	**	Calcium/calmodulin-dependent protein kinase kinase 2
Camkk2(S495);	*	Calcium/calmodulin-dependent protein kinase kinase 2
Calm1 seq: 96 - 107 no mod	*	Calmodulin
Calm1 seq: 92 - 107 no mod	*	Calmodulin
Cdc42 seq: 154 - 163 no mod	*	Cell division control protein 42 homolog
Cdc42ep4(S116);	**	Cdc42 effector protein 4
Cdc42bpa(S1638);	*	Serine/threonine-protein kinase MRCK alpha
Rac3 seq: 154 - 163 no mod	*	Ras-related C3 botulinum toxin substrate 3
Stat3 seq: 181 - 197 no mod	***	Signal transducer and activator of transcription 3
Stat3(Y705);	*	Signal transducer and activator of transcription 3

Supplementary Figure 5. GSEA of metabolic pathways and analysis of chemokine expression levels and differential signaling in malignant cells after exposure to CAF-FAK-depleted CM.

(a) GSEA identifies metabolic pathways that are significantly upregulated in epithelial cells in human breast cancers with low stromal FAK when compared with epithelial cells in breast cancers with high stromal FAK using KEGG (Kyoto Encyclopedia of Genes and Genomes) database. Data from Finak *et al* dataset. **(b-c)** Images of whole Proteome cytokine arrays at high exposure **(b)** or low exposure **(c)**. Differentially regulated cytokines are highlighted. Images of dots used in the main figure are highlighted in red boxes. N= 2 separate arrays. **(d)** qRT-PCR reveals enhanced *Ccl7* and *Ccl8* transcription in FAK-depleted CAF cells; Bar charts show mean mRNA expression ± s.e.m. n=2 WT and 4 FAK-depleted CAF preps, 6 WT and 12 FAK-depleted technical repeats. **(e)** *CCL7* expression is inversely correlated with stromal FAK expression in breast cancer patients. **(f)** Representative images of protein profiler arrays for Ccl6, Ccl12, Ccl11 and Pentraxin 3 from WT-CAFs isolated from *MMTV+;FSP-Cre-;FAK*$^{fl/fl}$ mammary tumours treated with vehicle (DMSO) or 5 µM FAK inhibitor for 24 hours. Bar charts show fold change in cytokine expression levels compared to vehicle controls ± s.e.m. n=4 individual dots/treatment. **(g)** qRT-PCR analysis reveals that *Ccl6* and *Ccl12* transcript levels are enhanced in FAK-depleted CAFs. *Ccl6*, but not *Ccl12*, transcription is upregulated in WT-CAFs after treatment with FAK kinase inhibitor; *Ccl12* levels are reduced in FAK-depleted CAFs following PI3K inhibitor (GDC-0941) treatment; Bar chart shows mean mRNA expression ± s.e.m. n=6 technical repeats from two independent cell preparations. **(h)** qRT-PCR validation of siRNA knockdown of *Ccr1* and *Ccr2* in E0771 cells; Bar chart shows mean *Ccr1* mRNA expression ± s.e.m. n=3 technical repeats/treatment. **(i)** Phosphoproteomics analysis identifies enhanced phosphorylation of CTNNA1 and CTNNB1 supporting beta-catenin transactivation; changes in CaMKK2 phosphorylation, total calmodulin levels and Rho family of GTPases indicating activated GPCR signalling; together with increased total STAT3 and pSTAT3 expression. **P*<0.05, ***P*<0.01,****P*<0.001,*****P*<0.0001, nsd, no significant difference. Statistical analysis, two-sided Student's t-test.

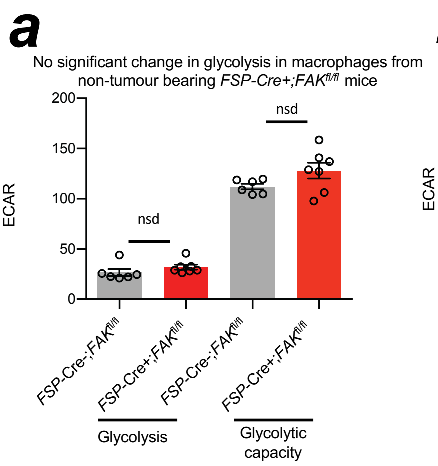

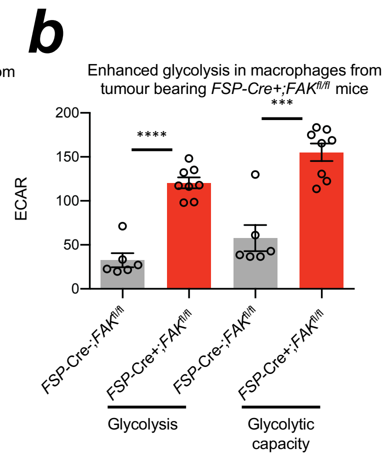

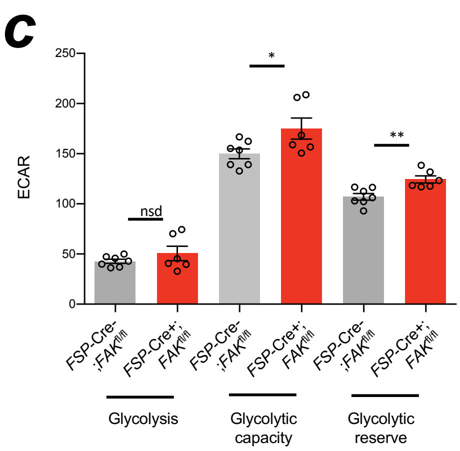

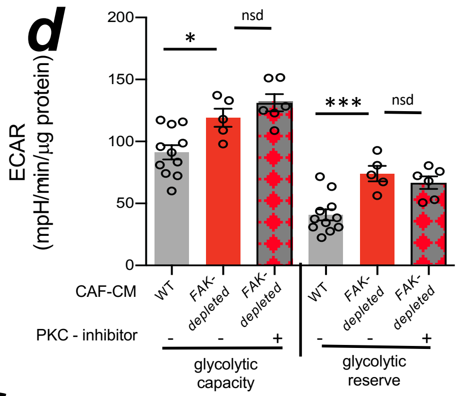

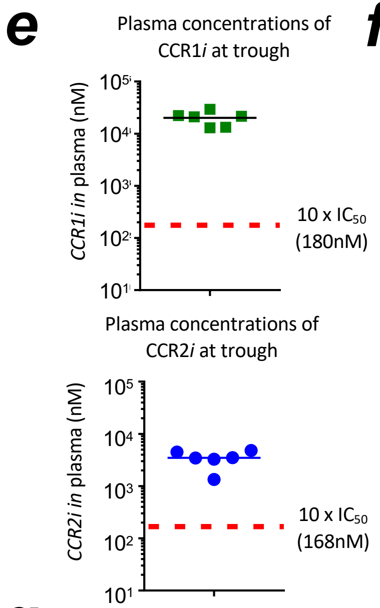

f

CCR2*i* is Highly Selective Against Other Chemokine and Chemoattractant Receptors

Receptor	Potency [nM]
CCR1	0.2
CCR2	>10,000
CCR3	~1,000
CCR4	>10,000
CCR5	>10,000
CCR6	>10,000
CCR7	>10,000
CCR8	>10,000
CCR9	>10,000
CCR10	>10,000

Receptor	Potency [nM]
CXCR1	>10,000
CXCR2	>10,000
CXCR3	>10,000
CXCR4	>10,000
CXCR5	>10,000
CXCR6	>10,000
C3aR	>10,000
C5aR	>10,000
FPRL1	>10,000

Receptor	Potency [nM]
CCR1	3,600
CCR2	2
CCR3	>5,000
CCR4	2,000
CCR5	2,200
CCR6	>5,000
CCR7	>5,000
CCR8	>5,000
CCR9	2,600
CCR10	>5,000

Receptor	Potency [nM]
CXCR1	>5,000
CXCR2	>5,000
CXCR3	>5,000
CXCR4	2,300
CXCR5	>5,000
CXCR6	>5,000
C3aR	2,200
C5aR	>5,000
FPRL1	>5,000

g

PKA-inhibition, KT5720		PKC inhibition, Go6983	
GO:0032007; P: negative regulation of TOR signalling cascade	****	GO:0004435; F:phosphatidylinositol phospholipase C activity	****
GO:0007188; P:G protein signalling, coupled to cAMP nucleotide second messenger	****	GO:0035025; P: positive regulation of Rho protein signal transduction	****
GO:0043408; P: regulation of MAPK cascade	****	GO:0070374; P: positive regulation of ERK1 and ERK2 cascade	****
GO:0007265; P: Ras protein signal transduction	****	GO:0014068; P: positive regulation of phosphatidylinositol 3-kinase cascade	****

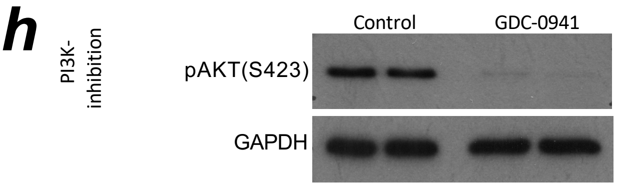

Supplementary Figure 6. Assessing effects on BMDMs from *FSP-Cre;FAK$^{fl/fl}$* mice, effect of PKC-inhibitor and efficacy and specificity of pharmacological inhibitors.

(**a**) BMDMs from non-tumour bearing *FSP-Cre+; FAK$^{fl/fl}$* mice show no changes in glycolysis or glycolytic capacity when compared with macrophages isolated from non-tumour bearing *FSP-Cre-; FAK$^{fl/fl}$* mice, n=3 independent experiments, scatter dots represent n=6 *FSP-Cre-;FAK$^{fl/fl}$* , n=7 *FSP-Cre+;FAK$^{fl/fl}$* technical repeats from the representative run. (**b**) BMDMs from tumour-bearing *MMTV+; FSP-Cre+; FAK$^{fl/fl}$* mice show enhanced glycolysis and glycolytic capacity when compared with macrophages isolated from tumour bearing *MMTV+; FSP-Cre-;FAK$^{fl/fl}$* mice, n=3 independent experiments, scatter dots represent n=6 *FSP-Cre-;FAK$^{fl/fl}$*, n=8 *FSP-Cre+;FAK$^{fl/fl}$* technical repeats from the representative run. *****P*< 0.0001. Two-sided Students t-test. (**c**) BMDMs from *MMTV+; FSP-Cre-; FAK$^{fl/fl}$* mice show enhanced glycolytic capacity and glycolytic reserve after treatment with FAK-depleted CAF CM. n=3 independent experiments, scatter dots represent n=7 *FSP-Cre-;FAK$^{fl/fl}$* , n= 6 *FSP-Cre+;FAK$^{fl/fl}$* technical repeats from the representative run. (**d**) Extracellular flux analysis of breast epithelial cells exposed to WT or FAK-depleted CAF CM in the presence of PKC-inhibitor; n= 11 individual cell preps, exposed to WT CM, n=5 exposed to FAK-depleted CM, n=6 exposed to FAK-depleted CM + PKC inhibitor. Bar charts in **a**-**d** show mean ECAR ± s.e.m. (**e**) Evidence of *in vivo* efficacy of CCR1*i* and CCR2*i*. Tumour bearing *FSP-Cre+;FAK$^{fl/fl}$* mice were treated with CCR1*i* and CCR2*i* once daily from day 8 until the end of the study at day 34. Trough plasma levels, assessed on blood samples taken 24 hours after the last dose, were greater than 10 x IC_{50} (see dotted red line) for each compound, resulting in complete CCR1 and CCR2 receptor engagement by CCR1*i* and CCR2*i*, respectively; Measure of centre shows the mean CCR1*i* plasma concentration (nM) from n=6 blood samples from both CCR1*i* and CCR2*i* treated *FSP-Cre+;FAK$^{fl/fl}$* mice. (**f**) CCR1*i* and CCR2*i* have high specificity for their respective targets. Potency results are IC_{50} or A_2 values from different assays (binding, calcium flux, or migration) in buffer or serum using cells that express tested receptors. (**g**) TB32048 tumour cells were treated with PKA inhibitor (KT5720) or PKC inhibitor (Go6983) and mass spectrometry performed. Results showed significant alterations in several relevant downstream signaling pathways. *****P*<0.0001. (**h**) Primary fibroblasts were treated with PI3K inhibitor, GDC-0941, and Western blot analysis showed a reduction in pAKT (s473). GAPDH acts as a loading control. N= 2 independent cell preps. **P*<0.05, ***P*<0.01, ****P*<0.001, nsd, no significant difference. Statistical analysis, two-sided Student's t-test.

Supplementary Figure 7 Uncropped scans from Supplementary Fig. 2 , Demircioglu *et al.*

2c

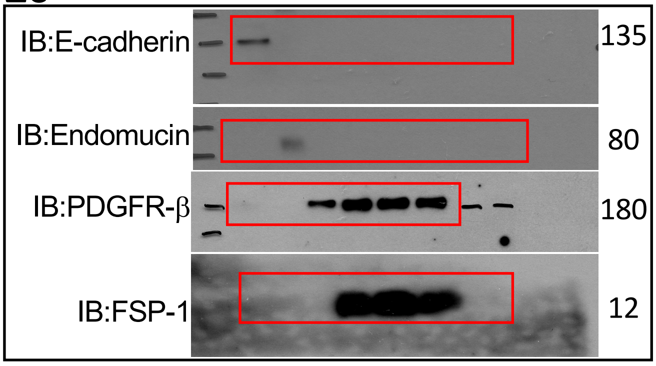

2d

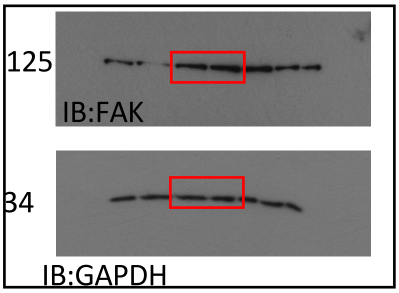

2f

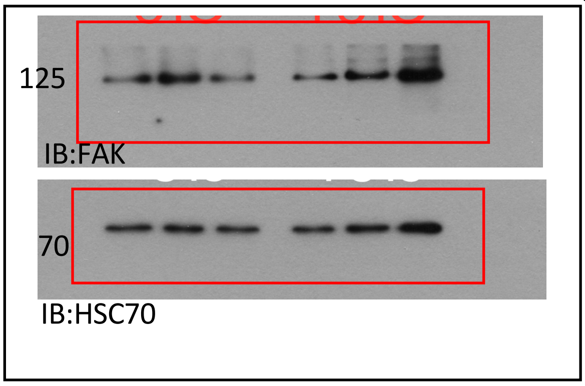

2g

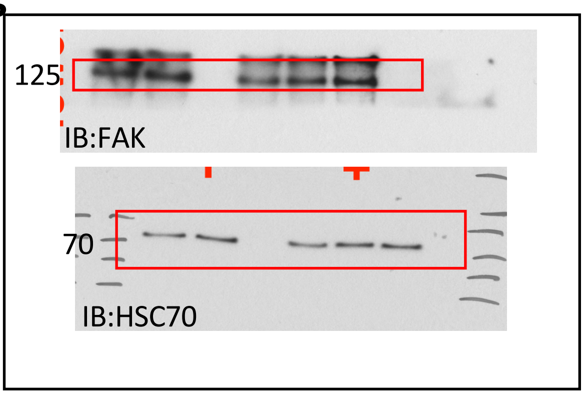

2h

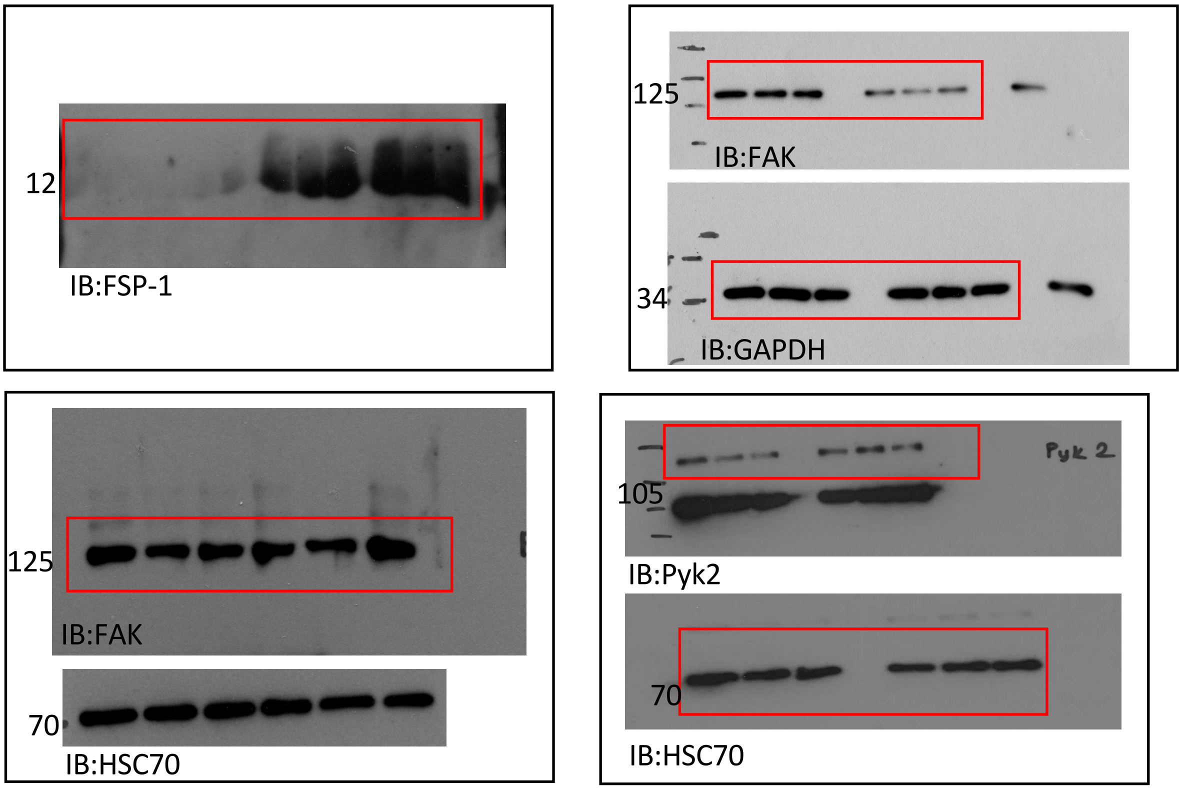

2i

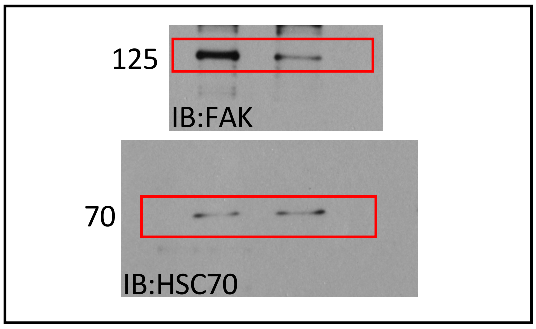

Uncropped scans from Supplementary Fig. 6h

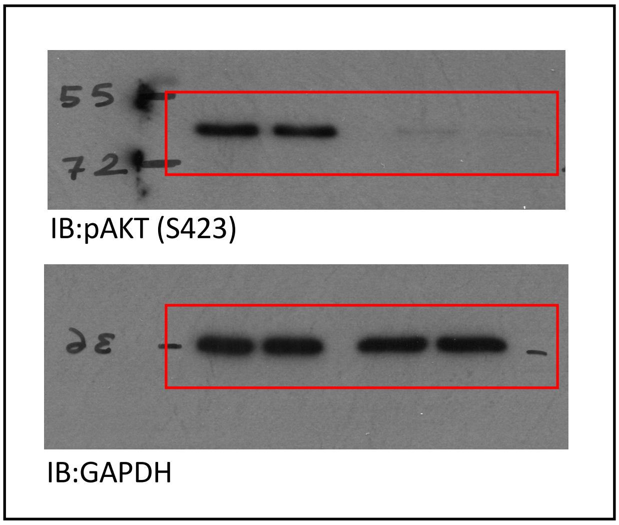

Supplementary Figure 7. Uncropped scans from Western blots.

a

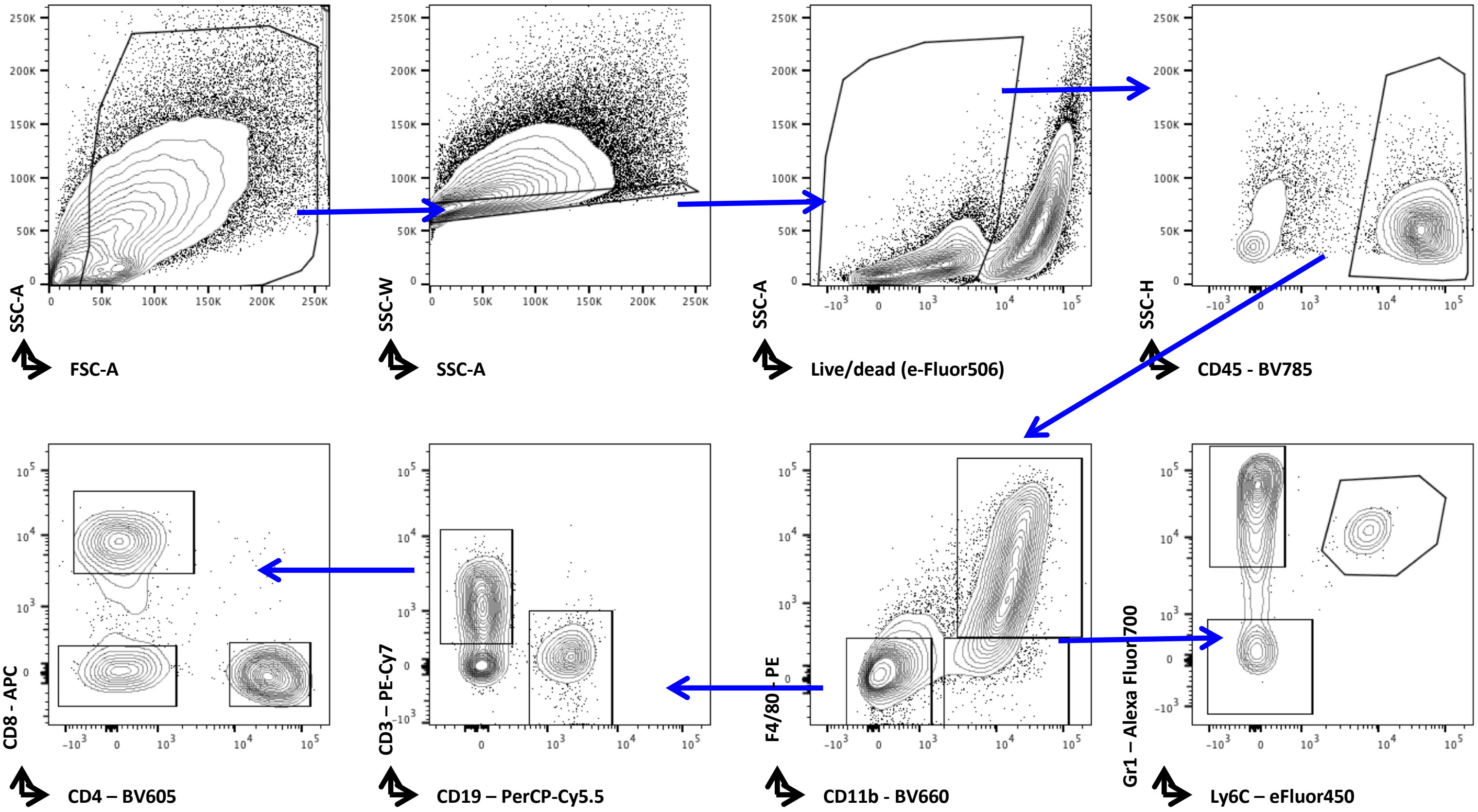

b

Cells gated on either CD4+ or CD8+ T cells. Here showing CD4+ cells

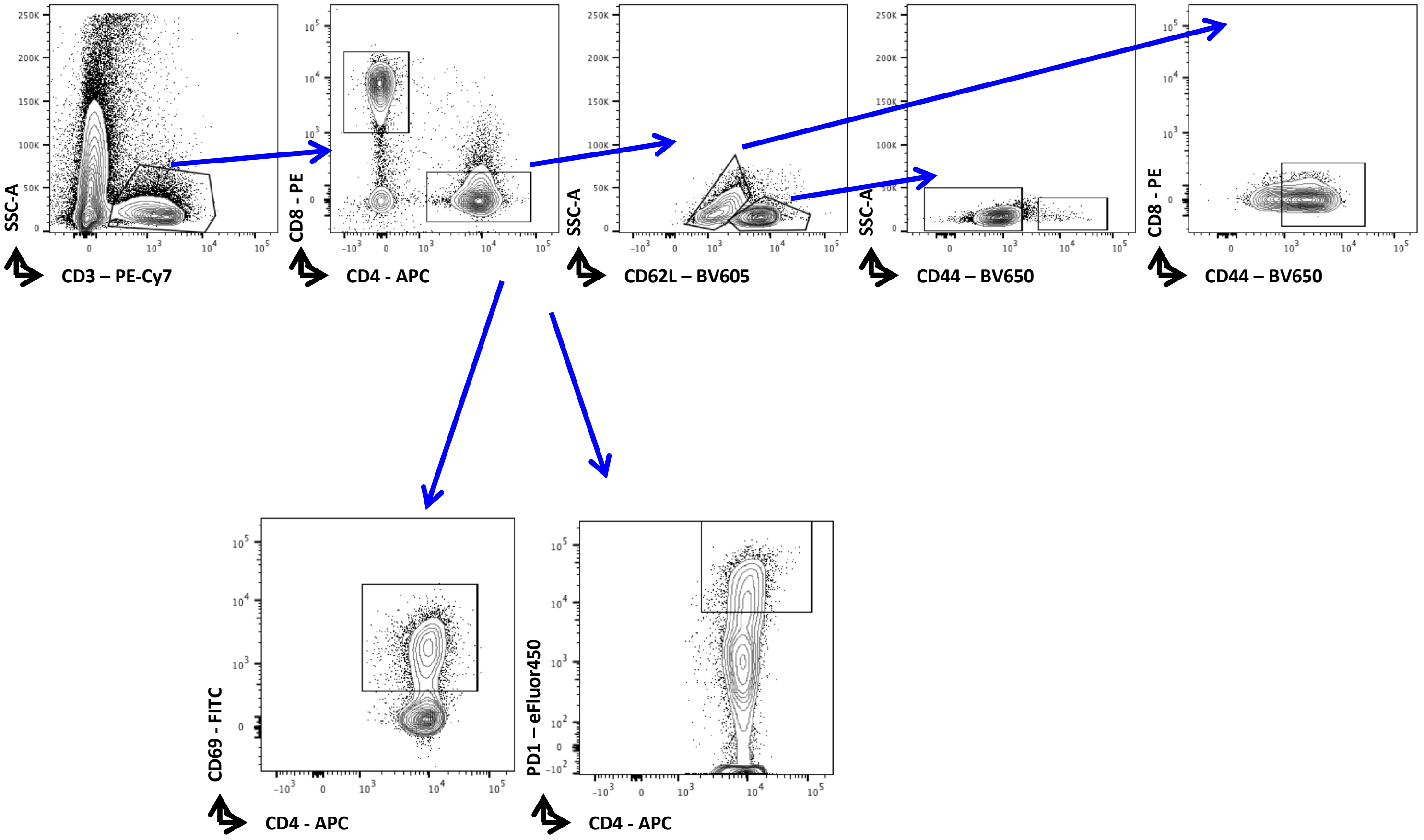

Supplementary Figure 8. Gating strategy for FACs analysis. (**a**) Gating strategy for identification of leukocytes by flow cytometry. Shown are representative images from the analysis of cells from orthotopic breast tumours. TAMs are defined as CD45+CD11b+F4/80+; Gr1+ cells as CD45+CD11b+F4/80-Gr1+,Ly6C+, CD19+; B cells as CD45+CD11b-F4/80-CD3-CD19+; CD4 T cells as CD45+CD11b-F4/80-CD19-CD3+CD4+ and CD8 T cells as CD45+CD11b-F4/80-CD19-CD3+CD8+. (**b**) Gating strategy for identification of T cell markers: CD44, CD62L, CD69 and PD1.
