# Supplementary material for: Cancer associated fibroblast FAK regulates malignant cell metabolism
Source: Nat Commun. 2020 Mar 10;11:1290. doi: 10.1038/s41467-020-15104-3 (PMC7064590; doi:10.1038/s41467-020-15104-3)
Supplement: Supplementary file 2 — Reporting Summary [file 41467_2020_15104_MOESM2_ESM.pdf]

nature research

Corresponding author(s): K.HDOIVALA-DILKE

Last updated by author(s): Jan 14, 2020

Reporting Summary

Nature Research wishes to improve the reproducibility of the work that we publish. This form provides structure for consistency and transparency in reporting. For further information on Nature Research policies, see Authors & Referees and the Editorial Policy Checklist .

Statistics

For all statistical analyses, confirm that the following items are present in the figure legend, table legend, main text, or Methods section.

n/a	Confirmed	
☐	☒	The exact sample size (*n*) for each experimental group/condition, given as a discrete number and unit of measurement
☐	☒	A statement on whether measurements were taken from distinct samples or whether the same sample was measured repeatedly
☐	☒	The statistical test(s) used AND whether they are one- or two-sided<br>*Only common tests should be described solely by name; describe more complex techniques in the Methods section.*
☒	☐	A description of all covariates tested
☒	☐	A description of any assumptions or corrections, such as tests of normality and adjustment for multiple comparisons
☐	☒	A full description of the statistical parameters including central tendency (e.g. means) or other basic estimates (e.g. regression coefficient) AND variation (e.g. standard deviation) or associated estimates of uncertainty (e.g. confidence intervals)
☒	☐	For null hypothesis testing, the test statistic (e.g. *F*, *t*, *r*) with confidence intervals, effect sizes, degrees of freedom and *P* value noted<br>*Give P values as exact values whenever suitable.*
☒	☐	For Bayesian analysis, information on the choice of priors and Markov chain Monte Carlo settings
☒	☐	For hierarchical and complex designs, identification of the appropriate level for tests and full reporting of outcomes
☒	☐	Estimates of effect sizes (e.g. Cohen's *d*, Pearson's *r*), indicating how they were calculated

Our web collection on statistics for biologists contains articles on many of the points above.

Software and code

Policy information about availability of computer code

Data collection

For gene expression profiling (GEP) datasets:
Published data sets for breast cancer (Finak et al., 2008) and pancreatic cancer (Stratford et al 2010) were obtained from Gene Expression Omnibus (GEO) under the accession number of GSE9014 and GSE21501, respectively.
Data analysis
ImageJ software (V1.51) was used to quantify Western blot images and Picrosirius red staining
Images obtained from spinning disc were processed and analysed using Fiji
Axiovision Rel 4.9.1 software was used to capture IF images on the Zeiss Axioplan microscope and RNAscope images
FloJo software (V 10.0.8 tree Star) was used to analyse FACS data
MRI images were acquired using VivoQuant 3.0
Respiratory gating was done using ParaVision Acquisition 5.1
PET images were acquired with Inveon Acquisition Workplace software version 1.5
Mass spec data was analysed with XCalibur Qual Browser and XCalibur Quan Browser software (V3.0)
qRT-PCR data was analysed using StepOne Real Time PCR machine and software
Molecular Signatures Database (v6.0) and Enrichment Map was used for pathway analysis
Prism 7 software was used for statistical analysis of data

Data analysis

GEP data analysis:
All data analysis was performed in the R programming environment. Differential expression analysis was performed using limma. Sample clustering using the stroma gene signature in the pancreatic cancer data set was performed using non-negative matrix factorisation (NMF) consensus clustering. Survival analysis was performed using the 'survival' R pacakge. Pathway analysis was conducted using the prerank GSEA module in the GenePattern web application, followed by network construction using the Cytoscape network visualization software, Enrichment Map.

Proteomics:
Peptide identification and quantification was carried out from the MS/MS and MS data using the Mascot search engine. Kinase substrate enrichment analysis (KSEA) was carried out by grouping peptides into substrate sets known to be phosphorylated by a specific kinase. Gene ontology enrichment analysis of proteins differentially phosphorylated between conditions (at $p < 0.05$) was carried out using the hypergeometric test.

For manuscripts utilizing custom algorithms or software that are central to the research but not yet described in published literature, software must be made available to editors/reviewers. We strongly encourage code deposition in a community repository (e.g. GitHub). See the Nature Research guidelines for submitting code & software for further information.

Data

Policy information about availability of data

All manuscripts must include a data availability statement. This statement should provide the following information, where applicable:

- Accession codes, unique identifiers, or web links for publicly available datasets
- A list of figures that have associated raw data
- A description of any restrictions on data availability

I have included a Data availability statement at the end of the manuscript, before the refs.
“Data availability statement
For gene expression profilin (GEP), publicly available datasets were obtained and used, GSE9014 and GSE2150. The mass spectrometry proteomics data have been deposited to the ProteomeXchange Consortium via the PRIDE partner repository with the dataset identifier PXD008276 and 10.6019/PXD008276.
All the relevant data that support the findings of this study are available from the corresponding author on request."

Field-specific reporting

Please select the one below that is the best fit for your research. If you are not sure, read the appropriate sections before making your selection.

- [x] Life sciences
- [ ] Behavioural & social sciences
- [ ] Ecological, evolutionary & environmental sciences

For a reference copy of the document with all sections, see nature.com/documents/nr-reporting-summary-flat.pdf

Life sciences study design

All studies must disclose on these points even when the disclosure is negative.

Sample size	Samples sizes were determined according to power calculations. In consultation with our in-house statisticians, Prof Duffy and Dr North, we have done pilot studies to determine the numbers of animals required to provide statistical significance in our results. Prof Duffy has done the power calculations to estimate that we will require 10 mice/cohort. Using a two-sided test with a 5% significance level, his calculations predict 85-90% power to reject the null hypothesis of no difference assuming the two genotypes differ by the magnitudes that we have observed in similar experiments. For tumour growth and tumour response studies two-way ANOVA or Student's t-test will be used. For incidence of metastasis Chi-square, and for survival, Log-rank (Mantel-Cox) tests will be used.
Data exclusions	Inclusion criteria: within each experiment animal groups were the same age, sex, genetic strain and maintained under the same conditions. Exclusion criteria: mice were excluded from the experiment only if unexpected adverse effects were observed.
Replication	All experimental data are given including replicates. Details of experimental replicates are given in the figure legends. all reported attempts at replication were successful.
Randomization	Within each experiment, mice were randomly assigned to different groups to avoid bias. In other experiments cells were randomly assigned to groups to avoid bias. All histological analyses were done in a blinded fashion.
Blinding	All data collection and analysis was blinded.

Reporting for specific materials, systems and methods

We require information from authors about some types of materials, experimental systems and methods used in many studies. Here, indicate whether each material, system or method listed is relevant to your study. If you are not sure if a list item applies to your research, read the appropriate section before selecting a response.

Materials & experimental systems

n/a	Involved in the study	
[ ]	[x]	Antibodies
[ ]	[x]	Eukaryotic cell lines
[x]	[ ]	Palaeontology
[ ]	[x]	Animals and other organisms
[x]	[ ]	Human research participants
[x]	[ ]	Clinical data

Methods

n/a	Involved in the study	
[x]	[ ]	ChIP-seq
[ ]	[x]	Flow cytometry
[x]	[ ]	MRI-based neuroimaging

Antibodies

Antibodies used

Antibodies

Endomucin (clone V.7C7, Santa Cruz, cat no. sc-65495)
alpha-smooth muscle actin Cy3-conjugated (clone 1A4, Sigma-Aldrich, cat no. C6198)
AlexaFluor 488 goat anti-rabbit IgG (ThermoFisher Scientific, cat no. A-11008)
Ki67 (SP6, Abcam, cat no. ab16667)
OmniMap-anti rabbit HRP (Roche, cat no. 760-4311)
PE-PECAM (Clone 390, Biolegend, cat no. 102408)
HSC70 (Clone B6, Mouse monoclonal, Santa Cruz, cat no. sc7298)
GAPDH (clone 6C5, Mouse monoclonal, Millipore, cat no. MAB374)
Smooth muscle actin (Clone 1A4, Mouse monoclonal, DAKO, cat no. M0851)
FAK (Clone 4.47, Mouse monoclonal, Millipore cat no. 05-537)
FSP-1 (Rabbit polyclonal, Millipore cat no. 07-2274)
PDGFR- (Clone 28E1, Rabbit monoclonal, Cell Signaling cat no. 3169)
E-cadherin (Clone 24E10, Rabbit monoclonal, Cell Signaling cat no.3195)
Pyk2 (clone 5E2, Mouse monoclonal, Cell Signaling, cat no. 3480)
phospho-AKT (Ser473) (Clone 193H12, Rabbit monoclonal, Cell Signaling cat no. 4058)
phospho-p70 S6K (Thr389) (Rabbit polyclonal, Cell Signaling cat no. 9205)
CD45 Brilliant Violet 785™ (1 in 100 dilution, rat monoclonal, clone 30-F11, Biolegend, 103149)
CD3ε PE-Cy7 (1 in 50 dilution, Armenian hamster monoclonal, clone 145-2C11, Biolegend, 100320)
CD4 APC (1 in 200 dilution, rat monoclonal, clone RM4-4, Biolegend, 116014)
CD4 Brilliant Violet 605™ (1 in 200 dilution, rat monoclonal, clone RM4-4, Biolegend, 116027)
CD8 APC (1 in 200 dilution, rat monoclonal, clone 53-6.7, Biolegend, 100712)
CD8 PE (1in 200 dilution, rat monoclonal, clone 53-6.7, Biolegend, 100708)
CD69 FITC (1 in 100 dilution, Armenian hamster monoclonal, clone H1.2F3, Biolegend, 104506)
CD44 Brilliant Violet 650™ (1 in 100 dilution, rat monoclonal, clone IM7, Biolegend, 103049)
CD62L Brilliant Violet 605™ (1 in 100 dilution, rat monoclonal, clone MEL-14, Biolegend, 104438)
CD19 PerCP (1 in 200 dilution, rat monoclonal, clone 6D5, Biolegend, 115532)
CD11b Brilliant Violet 650™ (1 in 100 dilution, rat monoclonal, clone M1/70, Biolegend, 101259)
F4/80 PE (1 in 50 dilution, rat monoclonal, clone BM8, Biolegend, 123110)
PD1 eFluor® 450 (rat monoclonal, clone RMP1-30, eBioscience 48-9981-80)
anti-Gr1 Alexa Fluor® 700 (1 in 200 dilution, rat monoclonal, clone RB6-8C5, eBioscience, 56-5931-82)
anti-Ly6C eFluor® 450 (1 in 100 dilution, rat monoclonal, clone HK1.4, eBioscience 48-5932-82)
Fixable Viability Dye eFluor™ 506 (1 in 500 dilution, eBioscience, 65-0866-14)

Pimonidazole (Hypoxyprobe, cat no. HP-200mg)

Validation

All antibodies were validated by the commercial supplier except for the CCr1i/CCR2i antibody that was given to us as a gift as part of the academic collaboration with Chemocentryx. All validation statements are found on the respective antibody website.

Eukaryotic cell lines

Policy information about cell lines

Cell line source(s)

E0771 (from Prof. Anne Ridely, University of Bristol, UK) with the approval from F.M. Sirotnak (Memorial Sloan-Kettering Cancer Center, New York, USA).
TB32048 (Prof. David Tuveson, Cold Spring Harbour, USA).
WEHI-274, ATCC, cat no. CRL-1679

Authentication	The lines used were all derived from mouse. We have not authenticated these lines ourselves. See suppliers websites for authentication details.
Mycoplasma contamination	ALL CELLINES USED WERE MYCOPLASMA FREE
Commonly misidentified lines (See ICLAC register)	none as far as we know.

Animals and other organisms

Policy information about studies involving animals; ARRIVE guidelines recommended for reporting animal research

Laboratory animals

For animals bred in-house- health screens (quarterly) were conducted in accordance with FELASA guidelines for health monitoring of rodent colonies, to confirm their free statues of known pathogens in accordance with FELASA screens. No clinical signs were detected. Animals were housed in groups of 4-6 mice per individually ventilated cage in a 12 h light dark cycle (06:30-18:30 light; 18:30-06:30 dark), with controlled room temperature (21 ± 1 °C) and relative humidity (40-60 %). The cages contained 1-1.5 cm layer of animal bedding, and with environmental enrichment including cardboard Box-tunnel and crinkled paper nesting material. Animals had access to food and water ad libitum.

Species: Mouse.

Female FAK floxed mice (C57/BL6) were bred with FSP-1 Cre male mice (C57/BL6, originally developed by Prof Gustavo Leone, Cleveland Ohio, USA)1-3 to generate FSP-Cre-;FAKfl/fl and FSP-Cre+;FAKfl/fl mice. MMTV-PyMT+;FSP-Cre+;FAKfl/fl were also generated by crossing MMTV-PyMT+ mice with FSP-Cre+;FAKfl/fl mice.

In our study we used the FSP-1-Cre mice developed by Gustavo Leone, Cleveland Ohio, USA. These FSP-Cre+ mice display Cre activity specific in a subpopulation of activated fibroblasts and CAFs. FAK expression levels were not affected in epithelial cells and macrophages isolated from MMTV-PyMT+;FSP-Cre+;FAKfl/fl derived tumours indicating no effect in these cell types (Please see Extended figure 2 d, f and g).

To clarify, we have not used FSP-Cre mice from JaxLabs in this study. Although, JaxLabs also sell an FSP-Cre mouse line, these mice show poor specificity to fibroblasts or CAFs, and have also been shown to induce deletion of target genes in other cell types including epithelial cells and macrophages. The reason for the differences in the FSP-Cre+ transgenic from JaxLab and Leone's laboratory are likely to be related to the fact that they were generated independently by transgene insertion of different sequences of the FSP promoter regions.

Female FAK floxed mice (C57/BL6) were bred with FSP-1 Cre male mice (C57/BL6, originally developed by Prof Gustavo Leone, Cleveland Ohio, USA)1-3 to generate FSP-Cre-;FAKfl/fl and FSP-Cre+;FAKfl/fl mice. MMTV-PyMT+;FSP-Cre+;FAKfl/fl were also generated by crossing MMTV-PyMT+ mice with FSP-Cre+;FAKfl/fl mice.

In our study we used the FSP-1-Cre mice developed by Gustavo Leone, Cleveland Ohio, USA. These FSP-Cre+ mice display Cre activity specific in a subpopulation of activated fibroblasts and CAFs. FAK expression levels were not affected in epithelial cells and macrophages isolated from MMTV-PyMT+;FSP-Cre+;FAKfl/fl derived tumours indicating no effect in these cell types (Please see Extended figure 2 d, f and g).

To clarify, we have not used FSP-Cre mice from JaxLabs in this study. Although, JaxLabs also sell an FSP-Cre mouse line, these mice show poor specificity to fibroblasts or CAFs, and have also been shown to induce deletion of target genes in other cell types including epithelial cells and macrophages. The reason for the differences in the FSP-Cre+ transgenic from JaxLab and Leone's laboratory are likely to be related to the fact that they were generated independently by transgene insertion of different sequences of the FSP promoter regions.

Wild animals	This study did not involve wild animals
Field-collected samples	This study did not involve samples collected from the field
Ethics oversight	Queen Mary University of london. All animal work was carried out in accordance with ARRIVE Guidelines.

Note that full information on the approval of the study protocol must also be provided in the manuscript.

Flow Cytometry

Plots

Confirm that:

- [x] The axis labels state the marker and fluorochrome used (e.g. CD4-FITC).
- [x] The axis scales are clearly visible. Include numbers along axes only for bottom left plot of group (a 'group' is an analysis of identical markers).
- [x] All plots are contour plots with outliers or pseudocolor plots.
- [x] A numerical value for number of cells or percentage (with statistics) is provided.

Methodology

Sample preparation	Tumours were minced and incubated at 37°C for 20 min in an enzymatic digestion solution. For pancreatic TB32048 tumours, collagenase (clostridium histolyticum type V (2 mg/ml, Sigma-C9263)) in HBSS (Sigma) and for orthotopic E0771 and MMTV breast tumours, collagenase/dispase (1 mg/ml, Sigma-10269638001) in PBS was used to make a single cell suspension. DNase I (Sigma-DN25) was added into both digestion buffers, at a concentration of 0.5 mg/ml.
Instrument	BD LSRFortessa Cell Analyzer (BD Biosciences)
Software	Data were transferred and analyzed using the FlowJo software (Tree Star, Oregon, USA) version v10.0.8.
Cell population abundance	No cell sorting experiments were performed and thus cell population abundance is not relevant to our study
Gating strategy	Gate 1 (G1) was used to exclude debris (SSC-A versus FSC-A). After excluding debris, G2 was used to exclude doublets (SSC-W versus SSC-A). G3 was then used to select viable cells from the singlets (fixable viability dye negative cells (FVD - eFluor506) versus SSC-A). From viable cells gate 4 was used to select CD45+ cells (BV785 versus SSC-H).<br>Myeloid cells<br>For breast tumors, macrophages were defined as F4/80+ (PE) cells and for pancreatic tumors, macrophages were defined as CD11b+ (BV660);F4/80+.<br>F4/80- CD11b+ cells were gated and used to define Gr1+ (AlexaFluor700) cells. Gr1-Ly6C- (eFluor450) were gated and CD11c+ (FITC) cells defined as dendritic cells.<br>Lymphocytes<br>CD11b- cells were selected and used to define CD3+ (PE-Cy7) T cells and CD19+ B cells (PerCP-Cy5.5). From the CD3+ cells, T cell subsets were defined as CD4+ (BV605) T cells and CD8 (APC) T cells.<br>T cell activation markers<br>CD4+ (APC) and CD8 (PE) T cells were gated on CD3+ (PE-Cy7) cells and PD1+ (eFluor450), CD44 (BV650), CD62L (BV605) and CD69 (FITC) were used for activation markers.<br>Fluorescence minus one (FMO) controls were performed to help with setting up the gates.<br><br>Flow cytometric analysis of breast tumour tomato reporter mice. Debris, doublets and dead cells were excluded from the analysis using gates 1, 2 and 3 (G1-3) as described above. Viable cells (gate 3, G3) were selected and used to identify

☒ Tick this box to confirm that a figure exemplifying the gating strategy is provided in the Supplementary Information.
